# Prognostic significance and multivariate modeling of COL4A family genes and HMGA2 in glioma

**DOI:** 10.3389/fphar.2025.1591932

**Published:** 2025-04-25

**Authors:** Aijun Lu, Jiankun Zang, Na Tan, Liping Wei, Ying Liang, Zefeng Tan, Anding Xu, Dan Lu

**Affiliations:** ^1^ Department of Neurology and Stroke Center, The First Affiliated Hospital of Jinan University, Guangzhou, China; ^2^ Department of Neurology, The First People’s Hospital of Foshan, Foshan, China; ^3^ Clinical Neuroscience Institute, The First Affiliated Hospital of Jinan University, Guangzhou, China

**Keywords:** Glioma, type IV collagen, HMGA2, prognostic risk model, immune infiltration

## Abstract

**Background:**

COL4As, a group of six homologous genes that encode the type IV collagen α chains (α1-α6), have been identified as the main components of the collagen network in brain basement membranes. The distribution and generation changes of type IV collagen have been reported during glioma progression, but its underlying function of COL4As in glioma was still unclear.

**Methods:**

Based on the data of TCGA glioma cohort, we analyzed the correlation of COL4A family genes with the clinical characteristics and prognosis of glioma patients. By performing correlation and functional enrichment analysis, the interaction network of COL4As and their related genes in glioma were constructed to demonstrate the functional differences between COL4A members. By further screening the COL4As downstream factors, we sorted out the COL4As coregulated gene that could be the independent prognostic factor for glioma.

**Results:**

We found the high expressions in COL4A1 and COL4A2 were positively related to a worse prognosis of glioma patient, while, in COL4A3 and COL4A4 were predicted to a better prognosis. However, none of COL4As could function as an independent prognostic factor for glioma. HMGA2 is a coregulatory target of COL4A members through the COL4As-H19/HOTRAI-miR148a/miR222-HMGA2 axis. By being involved in the infiltration of Th2 cells and macrophages, HMGA2 could serve as an independent prognostic biomarker for glioma.

**Conclusion:**

In summary, our study revealed a potential common target of COL4A members HMGA2, which could serve as a novel prognostic factor for the diagnosis and therapy of glioma.

## 1 Introduction

Gliomas are the most common primary intracranial tumors, representing 81% of malignant brain tumors (Ostrom et al., 2014; Zhou et al., 2023), Diffuse infiltration into the surrounding brain parenchyma is a hallmark of most gliomas. Infiltrating glioma cells exist in close proximity with components of the tumor microenvironment, including the extracellular matrix (ECM) and infiltrating immune cells (Giordana et al., 1985; Mauro et al., 1984). While the levels of collagens in the normal adult brain are much lower than those in glioma (Niu et al., 2025; Kuang et al., 2023). Therefore, the interaction of tumor cells with basement membrane components is thought to be important in tumor invasive and metastatic properties (Liu et al., 2025). Type IV collagen (Col IV) is the main component of the collagen network in basement membranes (BMs). The pattern of Col IV distribution in normal brain tissues generally corresponds to the localization of basement membranes, such as the leptomeningeal membrane, pial-glial membrane, vascular endothelial cells, vascular smooth muscle cells, and Schwann cells (Kuang et al., 2023; Liu et al., 2025). However, Col IV is mainly confined to pial-glial membranes and thickened vessel walls in glioma (Katsuhiko et al., 1989). Col IV staining indicated that pial-glial membranes remained relatively intact and that the number of branching capillaries was significantly increased in low-grade glioma, while the disruption of pial-glial membranes and vascular glomeruloid proliferation was observed in highly invasive glioblastomas. Thus, the higher the grade of glioma, the higher the complexity of its vascular network, and the higher the degradation level of the pial-glial membrane (Tamaki et al., 1996). Col IV levels are markedly increased in gliomas, where it promotes extensive microvascular network formation. The microvascular network associated with its functional roles in key tumor progression processes including cell adhesion, migration, and angiogenesis. Consequently, Col IV is recognized as one of the crucial extracellular matrix proteins responsible for tumor growth and invasion. The network structure formed by Col IV likely serves as a major contributor to glioma progression, with increasing malignancy grade correlating with enhanced invasiveness, accelerated disease progression, and poorer clinical outcomes. The reason for this unbalanced Col IV distribution, in addition to the aggressiveness of high-grade glioma, may also be related to the types of Col IV. Col IV is generated by Col IV α chains which have six homologous members, i.e., α1, α2, α3, α4, α5 and α6 (Khoshnoodi et al., 2008), and encoded by the COL4A (type IV collagen α chain) family genes, including COL4A1, COL4A2, COL4A3, COL4A4, COL4A5 and COL4A6 (11). Previous studies have reported two kinds of Col IVs formed by the COL4As translation products at least. For example, the major Col IV composed of two α1 and one α2 chains is the major component of BMs; the minor Col IV composed of α3, α4 and α5 chains or two α5 and one α6 chains further constitutes the collagen network (Xiao et al., 2015). Minor Col IV has a much greater density of disulfide interchain crosslinks (Cosgrove and Liu, 2017) than major Col IV, which means that the collagen network formed by minor Col IV is more compact, stable and resistant to proteolytic degradation than that formed by major Col IV (Kalluri et al., 1997). The distribution of Col IVs has been proved has gradual change on glioma progression. But the unbalanced distribution of Col IV and underling function of COL4As family genes have not been reported yet, and which is a valuable issue worthy of further investigation.

**FIGURE 1:**
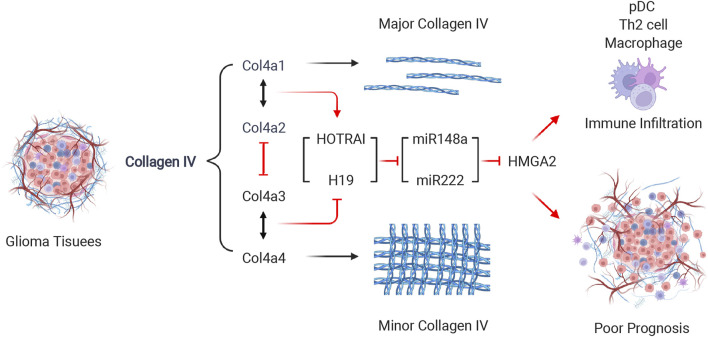
Graphical abstract. The key findings of study.

In the present study, we demonstrated the functional differences of each product of COL4As family genes, and screened their co-regulatory prognostic factors through functional difference analysis and interaction network construction in glioma, the key findings of this study are summarized in Figure 1. By conducting the survival analysis and establishing the prognostic model, we presented a novel COL4As related target for the diagnosis and treatment of glioma in future.

## 2 Materials and methods

The expression array data of six COL4A family members in pancancer were obtained from Oncomine datasets (https://www.oncomine.org) (Rhodes et al., 2007). The mRNA expression levels of COL4As in pancancer samples were compared with those in normal controls using Student’s t-test to generate a p value. The cutoffs of the p value and fold change were defined as 0.01 and 2, respectively. The clinical and TPM RNA Seq data of COL4As and the hub ceRNAs in glioblastoma (GBM) and brain low-grade glioma (LGG) were obtained from the TCGA datasets (http://cancergenome.nih.gov/) (Tomczak et al., 2015) and GTEx v.7 datasets in Xena Browser (https://xenabrowser.net/datapages/) (Chandrashekar et al., 2017). The other glioma RNA sequencing data were obtained from the GEO database (https://www.ncbi.nlm.nih.gov/gds/, GSE50161, GSE7696 and GSE4290) and CGGA database (http://cgga.org.cn/, mRNAseq_325 dataset) (Zhao et al., 2021). The collection data of glioma in this study were obtained from the online datasets, therefore additional ethics committee approval was not applicable. The analytical methods used in this study are presented in supplementary materials.

## 3 Results

### 3.1 Expression levels and functional enrichment analysis of COL4A family in patients with glioma

**FIGURE 2:**
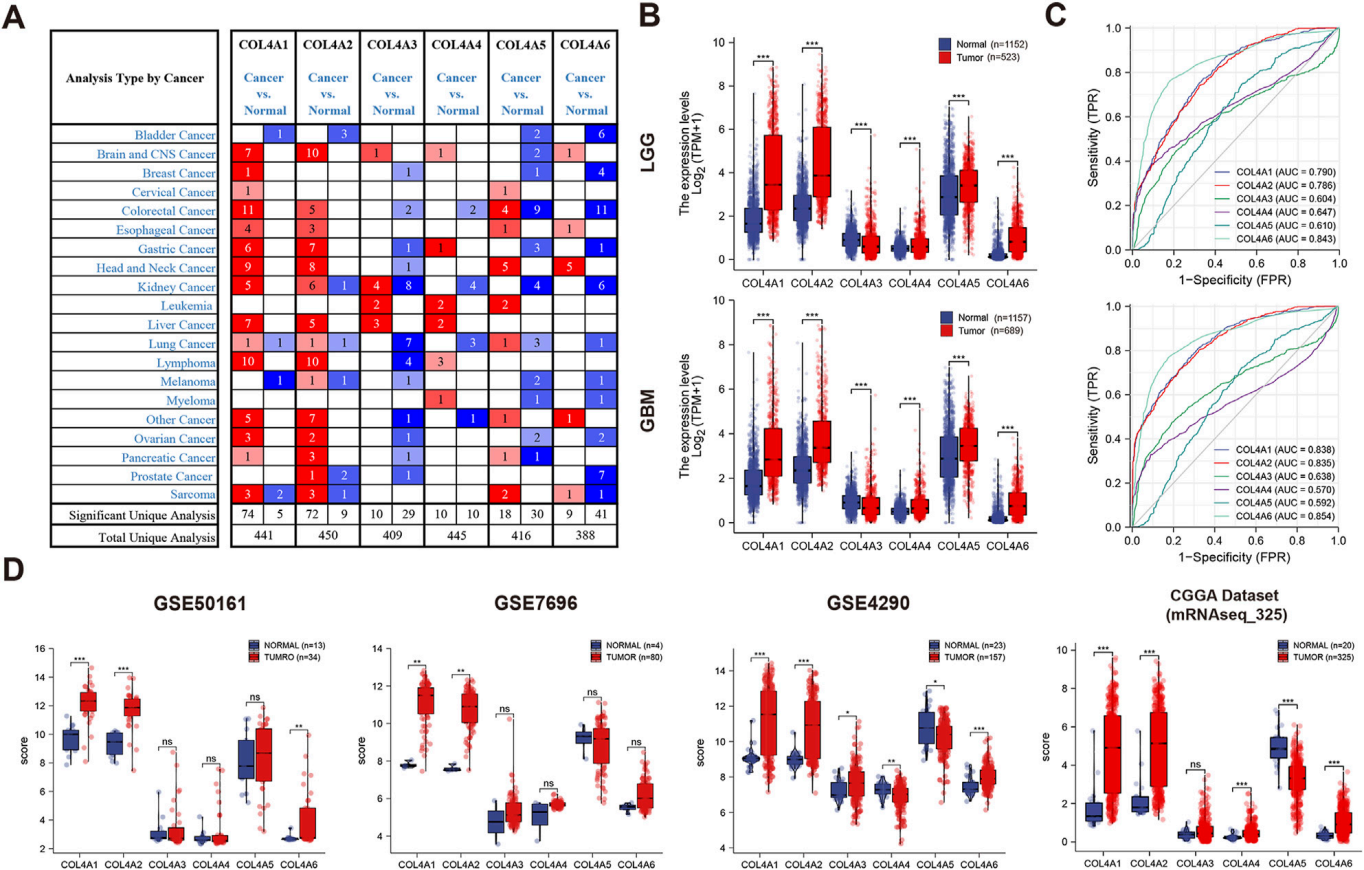
The transcription levels of COL4A family members in different types of cancers **(A)**. Expression level of COL4A members in normal and pancancer tissues **(B)**. Comparison of COL4A member expression in glioma tissues (LGG and GBM) and normal tissues in the TCGA dataset **(C)**. ROC analysis of the correlation of COL4A expression with glioma (LGG and GBM) **(D)**. Comparison of COL4A member expression in GBM tissues and normal tissues in three GEO datasets and one CGGA dataset.

Six members have been identified in the COL4A family of proteins. By using Oncomine databases, we found that the expression of COL4A1 to COL4A6 was closely related to brain and CNS cancer (Figure 2A). In TCGA, GEO (GSE50161, GES7696, GES4290) and CGGA (mRNAseq_325) databases, we found the COL4A1, COL4A2 and COL4A6 expression were significantly increased in glioma tissues. In contrast, the variation of COL4A3, COL4A4 and COL4A5 expression between tumor and normal tissues were lacking of significance, and presented an inconsistent tendency in three databases (Figures 2B, D). Similarly, as shown in Figure 1C and Tables 1, 2, the ROC curve analysis also indicated that the expression level of COL4A1, COL4A2 and COL4A6 has a higher correlation with GBM (AUC = 0.838, 0.835 and 0.854) and LGG (AUC = 0.790, 0.786 and 0.843) than that of COL4A3, COL4A4 and COL4A5 (AUC = 0.639, 0.570 and 0.592 in GBM; 0.604, 0.647 and 0.610 in LGG).

**TABLE 1:** The correlation of COL4A Family with clinical status in LGG patients.

Gene	Clinical status	Area under the curve (AUC)	95% confidence interval (CI)	Cut-off value	Sensitivity	Specificity
COL4A1	LGG Status (Nor vs. Tumo*)	0.790	0.767–0.812	2.021	0.772	0.658
COL4A2		0.786	0.764–0.809	2.760	0.740	0.678
COL4A3		0.604	0.571–0.636	0.580	0.442	0.793
COL4A4		0.647	0.616–0.678	0.820	0.388	0.909
COL4A5		0.610	0.582–0.638	2.798	0.748	0.473
COL4A6		0.843	0.822–0.865	0.328	0.763	0.818

* Nor, Normal, Tumo, Tumor.

**TABLE 2:** The correlation of COL4A Family with clinical status in GBM patients.

Gene	Clinical status	Area under the curve (AUC)	95% confidence interval (CI)	Cut-off value	Sensitivity	Specificity
COL4A1	GBM Status (Nor vs. Tumo*)	0.838	0.820–0.857	2.252	0.766	0.726
COL4A2		0.835	0.817–0.854	3.057	0.711	0.775
COL4A3		0.639	0.610–0.667	0.561	0.475	0.808
COL4A4		0.570	0.539–0.599	0.828	0.328	0.911
COL4A5		0.592	0.567–0.619	2.795	0.721	0.473
COL4A6		0.854	0.836–0.873	0.339	0.766	0.827

*Nor, Normal, Tumo, Tumor.

**FIGURE 3:**
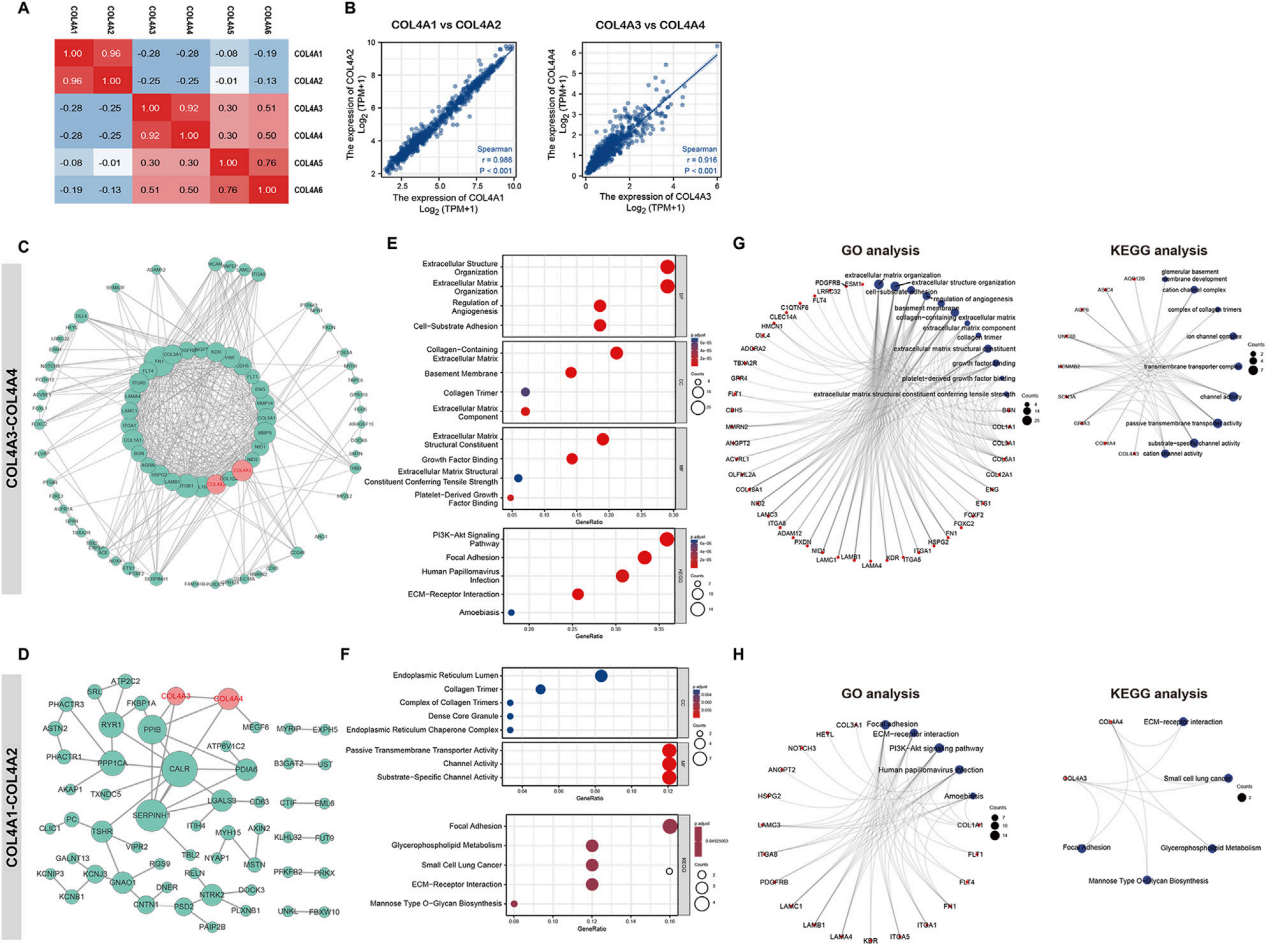
The correlation and interaction subnetwork between the COL4A family members **(A)**. The correlation analysis between the COL4A factors **(B)**. The correlation scatter plot for the COL4A-COL4A2 and COL4A3-COL4A4. The interaction network **(C)** and functional enrichment analysis **(E, G)** of COL4A1-2 and its associated genes. Interaction network **(D)** and functional enrichment analysis **(F, H)** of COL4A3-4 and its associated genes.

For another, by conducting the correlations analysis between COL4A family factors in Figure 3A, we found an obviously and positive relationship between the members in following two groups: COL4A1-COL4A2 (r = 0.96) and COL4A3-COL4A4 (r = 0.92), and also a negative correlation between the two groups of molecules (r ≤ −0.25). To explore the difference of these two pairs of factors, we first sorted out the 100 most frequently altered neighboring genes (co-expression in both GBM and LGG samples) for COL4A1-2 and COL4A3-4 by calculating the expression level (RNA Seq V2 RSEM) in the cBioPortal online database (GBM and LGG cohorts in TCGA, Firehose Legacy), and constructed the associated gene interaction networks for COL4A1-COL4A2 and COL4A3-COL4A4 (200 genes in each group). As shown in Figures 3B, C, after removing the unconnected genes, the network in COL4A1-CO4A2 was more abundant than that in COL4A3-COL4A4. The GO analysis indicated the extracellular matrix, angiogenesis, growth factor and cell adhesion were enriched in the COL4A1-COL4A2 network (Figure 3D); the endoplasmic reticulum, collagen and transport channels were enriched in the COL4A3-COL4A4 network. In KEGG analysis, excepted for the Focal Adhesion and ECM−Receptor Interaction were enriched in both the COL4A1-COL4A2 and COL4A3-COL4A4 networks, PI3K−Akt Signaling Pathway was enriched in the COL4A1-COL4A2 network, and Glycerophospholipid Metabolism and Mannose Type O−Glycan Biosynthesis were enriched in the COL4A3-COL4A4 network (Figure 3E); Therefore, we concluded that the two groups of factors were involved in the regulation of different biological functions during glioma progress, including angiogenesis and cell proliferation in COL4A1-COL4A2 functions, and cell invasion and communication in COL4A3-COL4A4 functions.

### 3.2 Association of the COL4As expression with the patients’ prognosis and clinicopathological parameters of glioma

**FIGURE 4:**
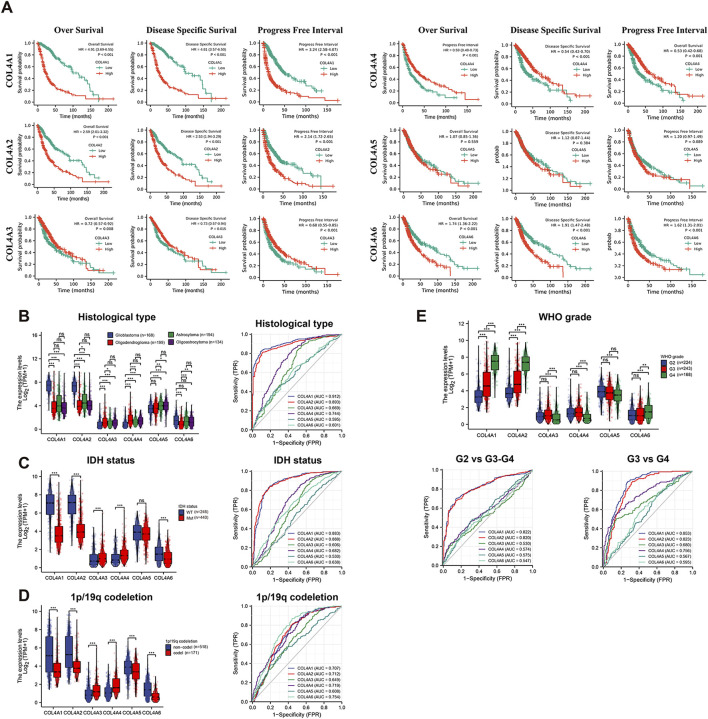
The prognostic value and association with clinical features of COL4A members in glioma **(A)**. The Kaplan-Meier curves analysis of COL4A factors in (GBM and LGG) patients. Correlation and ROC analysis of COL4As with histological type **(B)**, IDH status **(C)**, 1p/19q codeletion **(D)**, WHO grade **(E)** and primary therapy outcome **(F)** in glioma.

To explore the prognostic significance of COL4As in glioma, we used the Kaplan-Meier (K-M) curves to perform the survival analysis of COL4As in GBM and LGG cohorts from the TCGA datasets. As shown in Figure 4A, the high expression of COL4A1, COL4A2 and COL4A6 or the low expression of COL4A3 and COL4A4 can significantly increase the overall survival (OS), disease specific survival (DSS) and progress free survival (PFS) (*p* < 0.01) of GBM and LGG patients (Figure 4A). It indicated the COL4A1-2 were corelated to a worse prognosis, and COL4A3-4 predicted a better outcome in glioma. And the two groups of factors also presented an opposite effect on the glioma patients’ prognosis.

Meanwhile, the associations of COL4A expression with clinicopathological parameters in glioma patients were observed in Figures 4B–F (GBM and LGG samples from TCGA dataset). By comparing the expression level in different histological types of gliomas (Figure 3B; Table 3), COL4A1 and COL4A2 were better predictors of glioblastoma (AUC = 0.918 and 0.9), followed by COL4A3 and COL4A4 (AUC = 0.67 and 0.746). Meanwhile, the COL4A1 and COL4A2 have a negative correlation with IDH mutation status (AUC = 0.883 and 0.888) and 1p/19q deletion (AUC = 0.707 and 0.712), COL4A3 and COL4A4 have a positive correlation with IDH mutation status (AUC = 0.606 and 0.682) and 1p/19q deletions (AUC = 0.649 and 0.719). For another, the association between tumor grade and COL4A expressions were also analyzed in Figure 4E and Table 3. As the tumor progressed, COL4A1, COL4A2 and COL4A6 expression gradually increased, and COL4A3, COL4A4 and COL4A5 gradually decreased. The COL4A1 and COL4A2 had the most significant association with tumor grade (AUC = 0.822 and 0.820 in G2 vs G3 and G4; AUC = 0.863 and 0.834 in G3 vs G4), followed by COL4A3 and COL4A4 (AUC = 0.539 and 0.585 in G2 vs G3 and G4; AUC = 0.677 and 0.753 in G3 vs G4). Thus, this part of results further confirmed the COL4A1-2 were positively related to glioma progress, while COL4A3-4 have a negative effect on glioma development.

**TABLE 3:** The correlation of COL4A family with histological, IDH and 1p/19q mutation in GBM-LGG patients.

Gene	Clinical characteristics	Area under the curve (AUC)	95% confidence interval (CI)	Cut-off value	Sensitivity	Specificity
COL4A1	Histological (ABM and OABM and ODGBM vs. GBM)	0.918	0.898–0.939	4.063	0.82	0.917
	IDH (Mut vs. WT)	0.883	0.858–0.912	3.531	0.814	0.837
	1p/19q (non-codel vs. codel)	0.707	0.673–0.753	2.943	0.566	0.807
	Clinical stages (G3&G4 vs. G2)	0.822	0.790–0.855	2.942	0.703	0.857
	(G4 vs. G3)	0.853	0.828–0.898	4.063	0.917	0.708
	Primary therapy outcome (PD and SD vs. PR and CR)	0.647	0.597–0.698	3.63	0.61	0.616
COL4A2	Histological (ABM and OABM and ODGBM vs. GBM)	0.9	0.877–0.922	4.167	0.803	0.899
	IDH (Mut vs. WT)	0.888	0.866–0.916	3.295	0.768	0.874
	1p/19q (non-codel vs. codel)	0.712	0.680–0.761	3.258	0.552	0.819
	Clinical stages (G3&G4 vs. G2)	0.820	0.787–0.852	3.356	0.669	0.862
	(G4 vs. G3)	0.823	0.795–0.873	4.167	0.899	0.679
	Primary therapy outcome (PD and SD vs. PR and CR)	0.633	0.582–0.684	3.654	0.734	0.488
COL4A3	Histological (ABM and OABM and ODGBM vs. GBM)	0.67	0.618–0.721	0.189	0.814	0.512
	IDH (Mut vs. WT)	0.606	0.566–0.660	0.153	0.902	0.354
	1p/19q (non-codel vs. codel)	0.649	0.604–0.693	0.18	0.299	0.924
	Clinical stages (G3&G4 vs. G2)	0.530	0.494–0.584	0.121	0.2	0.942
	(G4 vs. G3)	0.680	0.622–0.731	0.189	0.512	0.827
	Primary therapy outcome (PD and SD vs. PR and CR)	0.506	0.453–0.558	1.412	0.328	0.744
COL4A4	Histological (ABM and OABM and ODGBM vs. GBM)	0.744	0.700–0.793	0.352	0.731	0.661
	IDH (Mut vs. WT)	0.682	0.648–0.736	0.378	0.743	0.61
	1p/19q (non-codel vs. codel)	0.719	0.679–0.763	0.381	0.473	0.871
	Clinical stages (G3&G4 vs. G2)	0.574	0.541–0.629	0.353	0.428	0.746
	(G4 vs. G3)	0.756	0.704–0.802	0.352	0.661	0.737
	Primary therapy outcome (PD and SD vs. PR and CR)	0.537	0.484–0.589	1.431	0.502	0.611
COL4A5	Histological (ABM and OABM and ODGBM vs. GBM)	0.595	0.552–0.646	2.463	0.455	0.714
	IDH (Mut vs. WT)	0.530	0.475–0.567	2.889	0.78	0.293
	1p/19q (non-codel vs. codel)	0.608	0.560–0.660	1.948	0.685	0.526
	Clinical stages (G3&G4 vs. G2)	0.587	0.542–0.633	1.91	0.406	0.75
	(G4 vs. G3)	0.565	0.509–0.620	2.647	0.786	0.354
	Primary therapy outcome (PD and SD vs. PR and CR)	0.522	0.470–0.575	3.562	0.452	0.66
COL4A6	Histological (ABM and OABM and ODGBM vs. GBM)	0.601	0.552–0.650	0.595	0.642	0.542
	IDH (Mut vs. WT)	0.638	0.589–0.677	0.595	0.68	0.537
	1p/19q (non-codel vs. codel)	0.754	0.722–0.797	0.461	0.6	0.842
	Clinical stages (G3&G4 vs. G2)	0.547	0.496–0.587	0.775	0.333	0.777
	(G4 vs. G3)	0.59	0.545–0.655	0.392	0.673	0.514
	Primary therapy outcome (PD and SD vs. PR and CR)	0.519	0.467–0.572	1.64	0.344	0.734

GBM, glioblastoma; ABM, astrocytoma; OABM, oligoastrocytoma; ODGBM, Oligodendroglioma; PD, progressive disease; SD, stable disease; PR, partial response; CR, complete respones

### 3.3 The COL4As related multiple-factors prognostic model for glioma

To investigate the prognostic value of COL4A members in glioma, we brought the TPM value of COL4As and clinical features into a stepwise Cox regression analysis (Supplementary Figure S1A, B). The result indicated that except for COL4A3 and COL4A5, the other COL4A1, COL4A2, COL4A4 and COL4A6 were significantly correlated with patient prognosis in univariate Cox regression analysis. However, in the further multivariate Cox regression analysis, none of the COL4A family members could function as an independent prognostic factor for glioma patients. Even so, by establishing the prognostic model in multivariate Cox regression analysis, we found that although none of the COL4A family members can function as the independent prognostic factors for glioma patients’ survival, the risk scores of this model still positively related to the expression of COL4A1 and COL4A2, and has a negative corelation with COL4A3 and COL4A4 (Supplementary Figure S1C).

Furthermore, the results in Supplementary Figure S1D, E indicated that the risk score of this model was significantly related to patients’ survival (HR = 6.17 in K-M analysis; AUC = 0.819 in ROC analysis). However, compared with the clinical prognostic model (including age, grade, 1p/19q codeletion, IDH status and histological type; HR = 6.13 in K-M analysis; AUC = 0.820 in ROC analysis), the prognostic efficiency has almost no difference between the two model in Decision Curve Analysis (DCA). Meanwhile, the clinical model seems to have a better performance than the risk model on 3- and 5-year survival (Supplementary Figure S1F). Therefore, we concluded that COL4A factors may have a potential impact on glioma patients’ survival, but not service as the risk factors directly.

### 3.4 Functional analysis of the COL4As related differentially expressed ceRNAs (DE-ceRNAs) and construction of the lncRNA-miRNA-mRNA triple regulatory network for the top DE-ceRNAs in glioma

**FIGURE 5:**
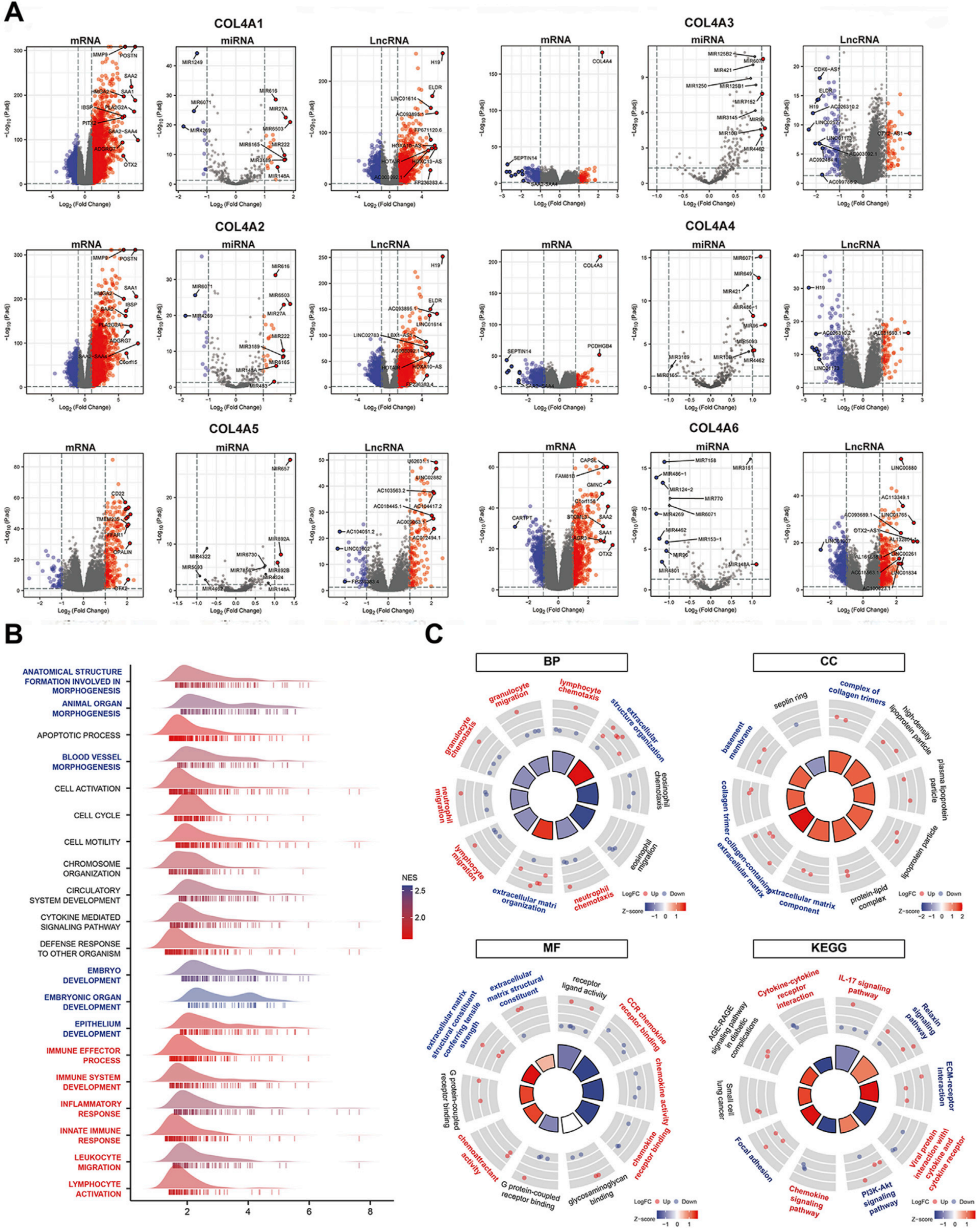
The identification and functional enrichment analysis of COL4As related DE-ceRNAs for glioma **(A)**. The volcano plots of the top 10 DE-ceRNAs between the COL4A high expression and COL4A low expression groups in glioma samples **(B)**. The GSEA analysis for the COL4As related differential expressed genes in glioma **(C)**. The GO and KEGG analysis for the top 10 differential expressed ceRNAs corelated to COL4As expression in glioma.

To further explore the mechanisms of COL4A factors involving in glioma progression, the DE-ceRNAs related to COL4As expressions were sort out from glioma sample in TCGA (including GBM and LGG). Then, the gene set enrichment analysis (GSEA) for the six sets of DE-mRNAs from the COL4Ashigh and COL4Aslow expression groups was performed in Figure 4B, and the result strongly implicated “tumor development (blue)” and “immune regulation (red)” as the main biological processes affected by COL4A factors. Secondly, we identified the top 10 DE-mRNAs, DE-miRNAs and DE-lncRNAs with the most significant changes in each COL4A member analysis were sorted by p < 0.05 and |logFC| >1 in Figure 5A (*p* < 0.05 and |logFC| >0.5 in miRNA analysis), and the heatmaps in Supplementary Figure S2 displayed the expression of the top 10 significantly variable genes in glioma samples with COL4Ashigh and COL4Aslow expression. Then, the GO and KEGG analysis of these factors was performed by R package. It showed that the 180 DE-ceRNAs were highly enriched in the biological processes of extracellular matrix (blue) and immune regulation (red) (Figures 5B, C).

**FIGURE 6:**
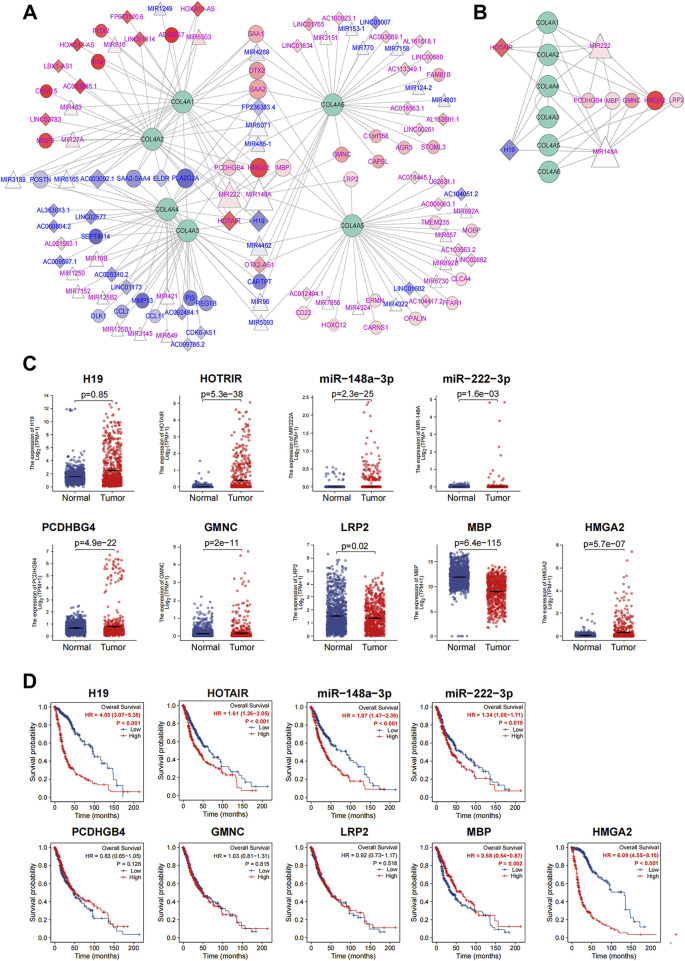
Construction of triple interaction network for COL4As related DE-ceRNAs and the analysis of the hub-factors in glioma (GBM and LGG) **(A)**. The triple interaction network for COL4As and the top differential expressed ceRNAs. The rhombus indicates lncRNAs; the triangle indicates miRNAs; and the circles indicate mRNAs. Blue represents downregulation, and red represents upregulation. The shade of the color represents the value of |logFC|. The size of the figure represents the connection degree of ceRNAs in the network **(B)**. The subnetwork of nine hub ceRNAs and COL4A members **(C)**. Comparison of hub DE-ceRNA expression between normal and tumor tissues **(D)**. Kaplan-Meier curve analysis of nine hub ceRNAs in glioma patients.

After that, we established the differential expression lncRNA-miRNA-mRNA triple regulatory network for COL4A family factors by using Cytoscape software (Figure 6A the diamond, triangle, and circle represent lncRNA, miRNA, and mRNA, respectively; the shades of color represent the value of fold changes, and the size of the shape represent the connection degree). Then, the ceRNAs with a connection degree ≥2 and correlated to at least one node of each different type of ceRNA were further selected to generate the hub-regulation network. It contained two lncRNAs (HOTAIR and H19), four miRNAs (miR222 and miR148a), and six mRNAs (PCDHGB4, MBP, GMNC, HGMA2 and LRP2) (Figure 6B).

### 3.5 The association of hub ceRNAs with COL4As and the construction of a hub ceRNA-associated prognostic model for glioma

To identify the crucial ceRNAs of great prognostic value in glioma, we compared the expression of hub ceRNAs from the triple regulatory network in tumor and adjacent normal tissues. We found that the expression of 9 DE-ceRNAs were significantly different between tumor tissues and normal tissues, including the upregulated H19, HOTAIR, miR222, miR148a, PCDHGB4, GMNC and HMGA2 and the downregulated LRP2 and MBP in tumors (Figure 6C). By comparing their correlations with COL4A factors, these ceRNAs had similar associations in COL4A1-COL4A2 and COL4A3-COL4A4, and only the H19, HOTRAI and HMGA2 were highly related to COL4As expressions (Supplementary Figure S3, *p* < 0.05 and |r| > 0.1). In addition, by performing K-M curve analysis (Figure 6D), the expression of H19, HOTAIR, miR-148a-3p, miR-222-3p, MBP and HMGA2 had a significant association with glioma patients’ survival. Thus, these COL4As-related ceRNAs may contribute to our glioma-specific prognostic model.

**FIGURE 7:**
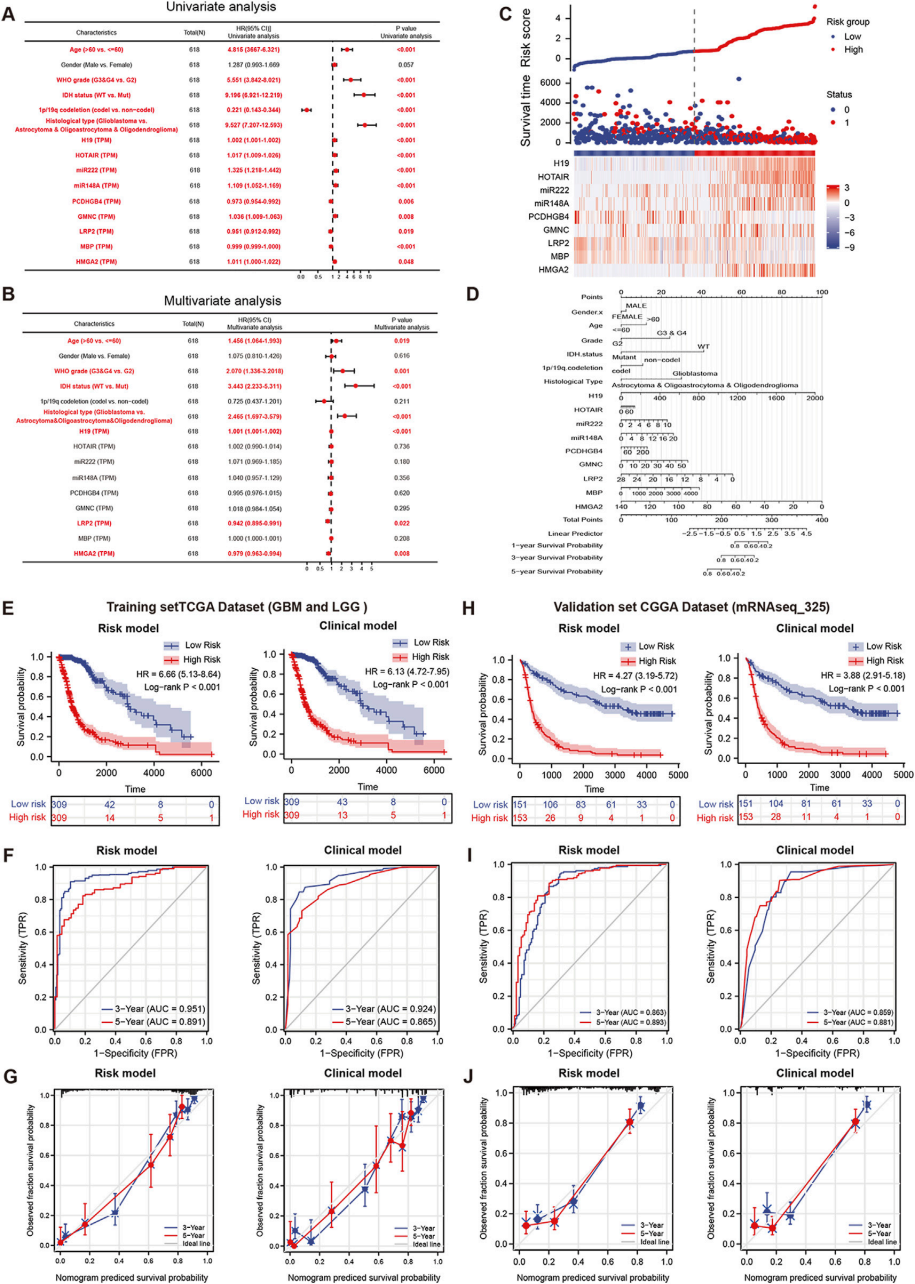
The prognostic model for the nine hub ceRNAs in glioma. Univariate **(A)** and multivariate **(B)** Cox regression analyses for clinical features and nine hub ceRNAs expression in the GBM and LGG cohorts **(C)**. The correlation between the nine hub ceRNAs expression and risk score in the prognostic model **(D)**. The nomogram for the prognostic model in Cox regression analysis. The efficiency comparation between the nine hub ceRNAs related risk model and clinical features model by K-M curves **(E)**, time dependent-ROC curve **(F)** and calibration curve **(G)** in the TCGA training cohorts. The efficiency comparation between the risk model and clinical features model by K-M curves **(H)**, time dependent-ROC curve **(I)** and calibration curve **(J)** in the CGGA validation cohort.

Combined with the clinical features, we further observed the prognostic significance of those ceRNAs by conducting Cox regression analyses (Figures 7A, B). The result indicated that except for the gender, 9 ceRNAs (including H19, HOTAIR, miR-2223p, miR148A, PCDHGB4, GMNC, LRP2, MBP and HMGA2) and 6 clinical features (age, grade, 1p/19q codeletion, IDH status and histological type) were closely related to OS (*p* < 0.05) in GBM and LGG cohorts. High expression levels of H19 (HR = 1.002, *p* < 0.001), HOTAIR (HR = 1.017, *p* < 0.001), miR-222 (HR = 1.325, *p* < 0.001), miR148A (HR = 1.109, *p* < 0.001), GMNC (HR = 1.036, *p* = 0.008) and HMGA2 (HR = 1.011, *p* = 0.048) were significantly associated with a worse prognosis, and the high expression of PCDHGB4 (HR = 0.0.973, *p* = 0.006) and MBP (HR = 0.951, *p* = 0.019) was significantly associated with a better prognosis in GBM and LGG patients (Figure 6A).

In multivariate analysis, the LRP2 (HR = 0.942, *p* = 0.22) and HMGA2 (HR = 0979, *p* = 0.008) were significantly associated with OS and may function as independent prognostic factors for GBM and LGG patients (Figure 7B). Then, based on the Cox regression analysis, the risk prognostic model was further conducted in Figure 7C, and a prognostic nomogram of this model is shown in Figure 7D. The risk scores of this model were calculated by the following formula:

To evaluate the performance of this risk model, we compared it with the clinical model (containing age, WHO grade, IDH status and histological type). The K-M curve analysis in Figure 7E indicated the risk model (left lane, HR = 6.66) has a better performance than the clinical model (right lane, HR = 6.13) in TCGA glioma cohort. The AUCs of 3-year survival and 5-year survival in ROC curve analysis showed a little improvement in risk model (Figure 7F, AUC = 0.951 and 0.891 in risk model; AUC = 924 and 0.865 in clinical model). And the calibration analysis of risk model also presented a better fitting degree than that of clinical model (Figure 7G).

For another, to further verify the predictive efficiency of the risk model, we performed external validation by using the CGGA dataset (mRNAseq_325). As shown in Figure 7H, the K-M curve analysis indicated the risk model (HR = 4.27) has a higher HR than that in clinical model (HR = 0.3.88). And the ROC curve of the 3-year survival and 5-year survival prediction in risk model (AUC = 0.863 and 0.893.) is better than clinical model (AUC = 0.859 and 0.881). The calibration analysis of risk model also indicated a higher fitting degree than that of clinical model. Therefore, this risk model is more accurate and efficient than the clinical parameters, and the ceRNAs concluded in this model may function as the critical factors in glioma progression.

### 3.6 HMGA2 is regulated by the COL4As-H19/HOTAIR-miR148a/miR222 axis and functions as a critical factor in glioma

**FIGURE 8:**
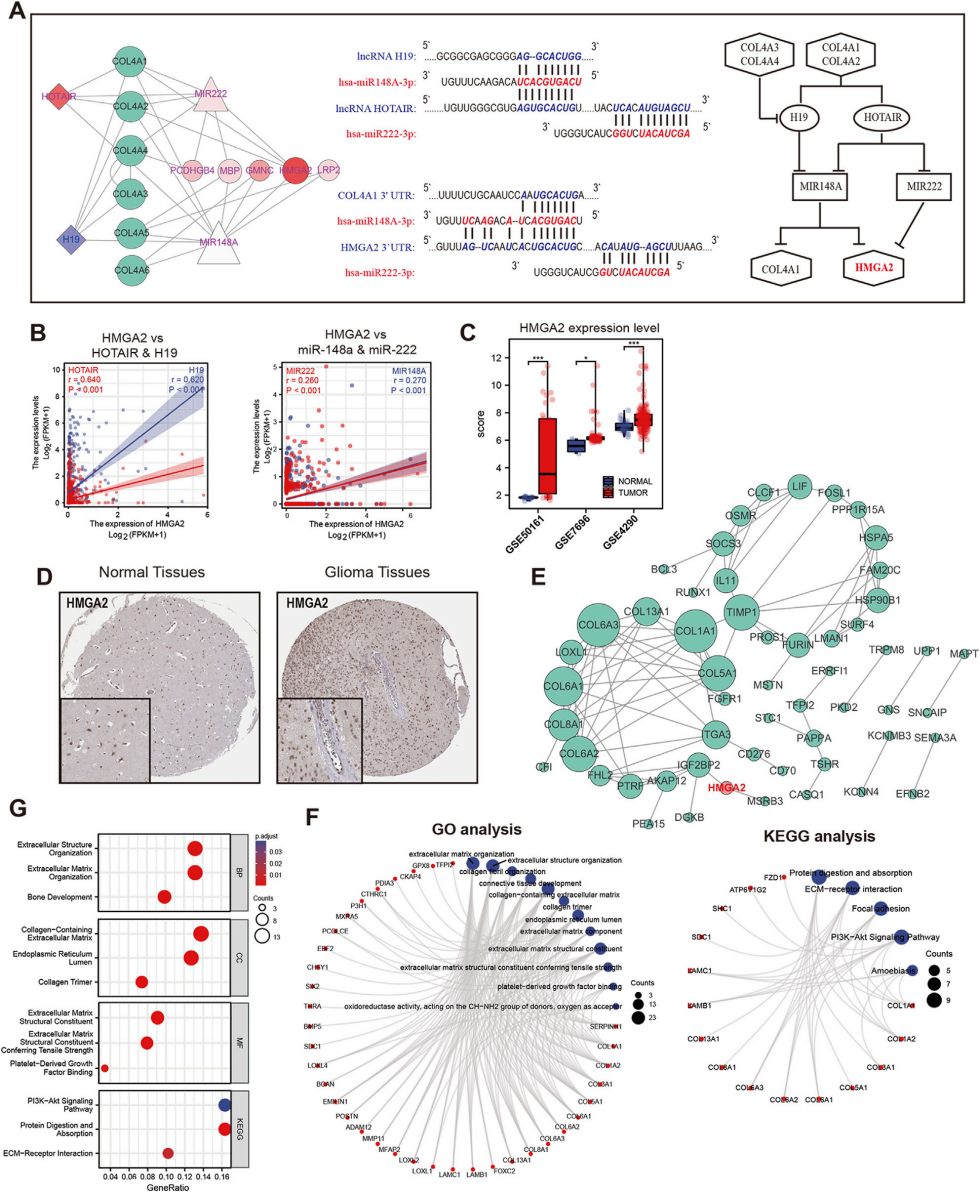
The functional analysis of the hub ceRNA regulatory network in glioma (GBM and LGG) **(A)**. Base pairing between miR-148a-3p/miR-222-3p and the target site in H19/HOTAIR and the 3′UTR of COL4A1/HMGA2 (left lane). The regulatory network between COL4As and hub ceRNAs (right lane) **(B)**. Correlation analysis of HMGA2 with H19 and HOTAIR and HMGA2 with miR222-3p and miR148a-3p in glioma **(C)**. Comparison of HMGA2 expression in GBM tissues and normal tissues in three GEO datasets **(D)**. Immunohistochemical staining for HMGA2 in normal and glioma tissues. The interaction network **(E)** and functional enrichment analysis **(F, G)** for HMGA2 and its related genes in glioma.

By using the online datasets LncBase Predicted v.2 and LncACTdb 2.0, we determined the base pairing between two miRNAs (miR-148a-3p and miR-222-3p) and target sites in lncRNA H19 and HOTAIR. Additionally, we predicted the binding sites of miR-148a-3p and miR-222-3p targeting the HMGA2 and COL4A1 3′UTRs by using the starBase v2.0, TarBase and miRbase databases (Figure 8A). Moreover, the expression correlation analysis in Figure 8B indicated that HMGA2 has a positive relationship with lncRNA H19 and HOTAIR expression and with miR-148a-3p and miR-222-3p. It indicated that HMGA2 may function as the co-target for COL4A factors and hub-ceRNAs network. For another, we further verified the expression of HMGA2 in different glioma cohort from GEO data sets (Figure 8C) and its distribution in tumor tissues (Figure 8D). Therefore, we drew the COL4As-H19/HOTAIR-miR148a/miR222-HMGA2 axis from the hub-ceRNAs network.

To explore the potential function of HMGA2, we identified the top 100 correlated genes enriched in both GBM and LGG and established a network (Figure 8E). By using GO and KEGG functional analysis, we found that HMGA2 and the related genes were enriched in collagen formation, ECM interaction (Figures 8F, G), which is highly consistent with the results in COL4A family. Therefore, HMGA2 was involved in the similar function with COL4A factors, and may service as a key factor for glioma progression.

### 3.7 Correlation between immune infiltration and expression of HMGA2 and COL4A family in glioma patients

Based on the function analysis in Figure 5, the COL4A factors and the related genes involved in both the “tumor development” and “immune regulation” processes. To identify the distribution and biological function of COL4As and HMGA2 more specifically, we used single-cell sequencing data from the GEO database to conduct dimensional-reduction clustering for the cells in GSE117891, and the cell enrichment of COL4As and HMGA2 were further analyzed. As shown in Supplementary Figure S4A, B, 5592 cells were sorted to perform clustering and were further divided into four groups (stromal or endothelial cells, immune cells, tumor cells and normal cells) by the related cell markers. The COL4A and HMGA2 enrichment analysis indicated that both of them were mainly expressed in tumor cells and immune cells (Supplementary Figure S4C, D). Due to the tumor-infiltrating lymphocytes are the independent predictors of sentinel lymph node status and patients’ survival in cancer (Azimi et al., 2012), we speculated that COL4As and HMGA2 may influence the patients’ prognosis by involving in the immune infiltration of glioma.

**FIGURE 9:**
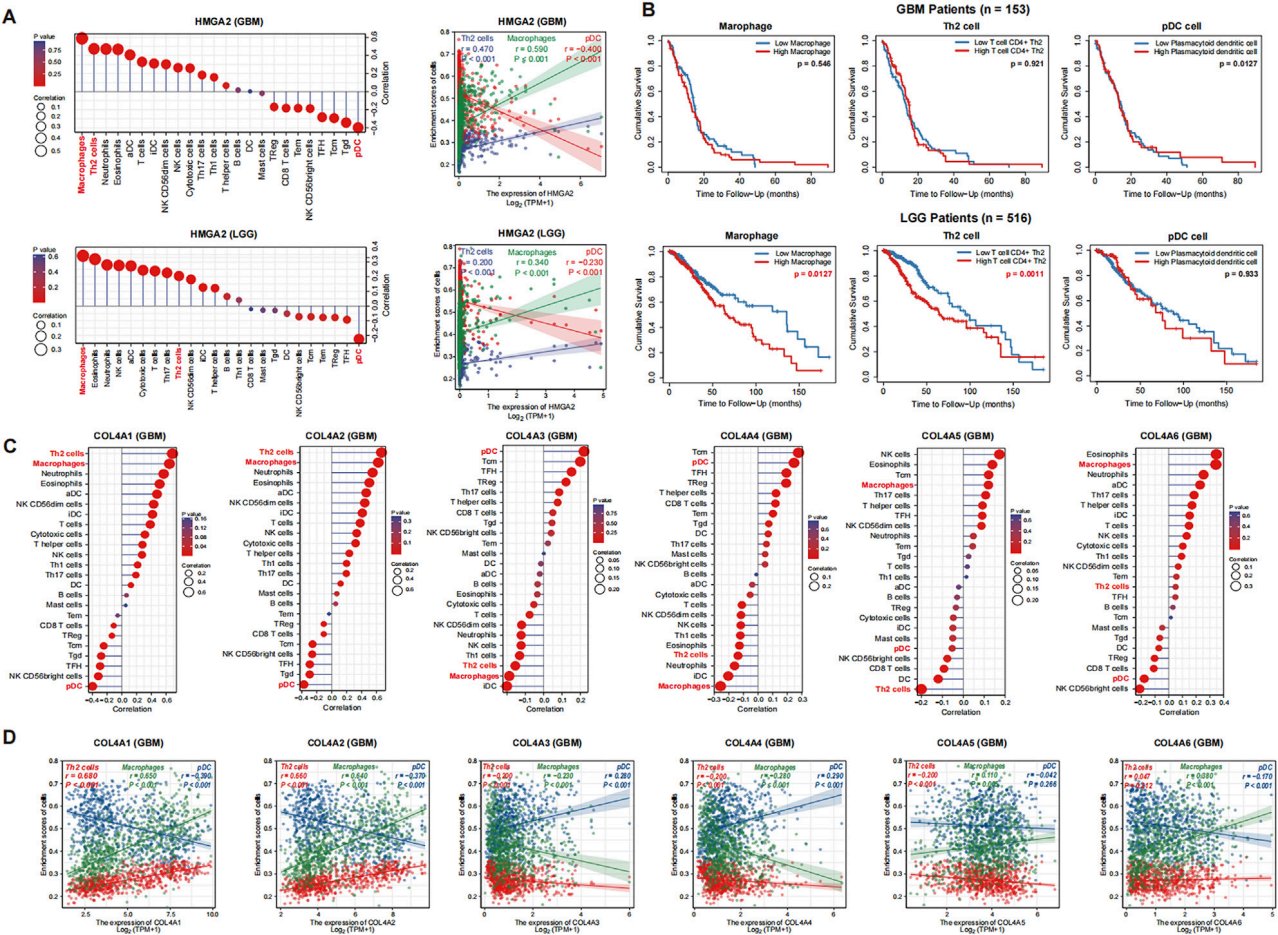
Correlation analysis of HMGA2 and COL4A immune infiltration in glioma (GBM and LGG) **(A)**. Association between HMGA2 gene copy number and immune cell infiltration levels in the GBM and LGG cohorts, respectively (left lane). Association analysis for HMGA2 with infiltration of the three immune cell types (right lane) **(B)**. Kaplan-Meier curve analysis of infiltration of three immune cell types in the GBM and LGG cohorts **(C)**. Association between COL4A member gene copy number and immune cell infiltration levels in GBM **(D)**. Association analysis for COL4As with infiltration of the three immune cell types in GBM.

To evaluate the potential effect of HMGA2 on immune infiltration in glioma, we conducted the correlation analysis of HMGA2 with various immune cell markers in glioma. The results indicated that several immune cell infiltration levels seemed to be associated with altered HMGA2 gene copy numbers in GBM (upper in Figure 9A; Table 4), including the two highest positive correlation cells: macrophages, Th2 cell infiltration (r = 0.589 and 0.47), and the highest negative correlation cell: plasmacytoid dendritic cell infiltration (pDC cells, r = −0.399) in GBM. Then, by using Kaplan–Meier curve analysis in the TIMER tool, we observed the infiltration of pDC cells and Th2 cell and macrophage effects on the OS of GBM patients. However, the infiltration of three immune cells had no significant effect on GBM patient prognosis (upper panel in Figure 9B; Table 5).

**TABLE 4:** Correlation analysis between HMGA2 and biomarkers of immune cells in GBM patients.

Genes	Cell type	Correlation (pearson)	P value (pearson)	Correlation (spearman)	P value (spearman)
HMGA2	Macrophages	**0.347**	**<0.001**	**0.589**	**<0.001**
HMGA2	Th2 cells	**0.340**	**<0.001**	**0.474**	**<0.001**
HMGA2	Neutrophils	0.243	<0.001	0.471	<0.001
HMGA2	Eosinophils	0.197	<0.001	0.467	<0.001
HMGA2	aDC	0.182	<0.001	0.406	<0.001
HMGA2	T cells	0.159	<0.001	0.329	<0.001
HMGA2	iDC	0.160	<0.001	0.311	<0.001
HMGA2	NK CD56dim cells	0.174	<0.001	0.302	<0.001
HMGA2	NK cells	0.226	<0.001	0.264	<0.001
HMGA2	Cytotoxic cells	0.126	<0.001	0.259	<0.001
HMGA2	Th17 cells	0.083	0.028	0.184	<0.001
HMGA2	Th1 cells	0.054	0.151	0.159	<0.001
HMGA2	T helper cells	0.088	0.020	0.067	0.076
HMGA2	B cells	−0.094	0.013	0.017	0.650
HMGA2	DC	−0.000	0.998	0.003	0.930
HMGA2	Mast cells	−0.079	0.037	−0.018	0.636
HMGA2	TReg	−0.049	0.197	−0.169	<0.001
HMGA2	CD8 T cells	−0.101	0.008	−0.183	<0.001
HMGA2	Tem	0.027	0.475	−0.183	<0.001
HMGA2	NK CD56bright cells	−0.125	<0.001	−0.187	<0.001
HMGA2	TFH	−0.282	<0.001	−0.283	<0.001
HMGA2	Tcm	−0.112	0.003	−0.293	<0.001
HMGA2	Tgd	−0.401	<0.001	−0.343	<0.001
HMGA2	pDC	**−0.286**	**<0.001**	**−0.399**	**<0.001**

Infiltration levels of macrophages and Th2 cells show strong positive correlations with HMGA2 expression in GBM, while pDC exhibit a strong negative correlation with HMGA2.

**TABLE 5:** Correlation analysis between HMGA2 and biomarkers of immune cells in LGG patients.

Genes	Cell type	Correlation (pearson)	P value (pearson)	Correlation (spearman)	P value (spearman)
HMGA2	aDC	0.153	<0.001	0.273	<0.001
HMGA2	Macrophages	**0.217**	**<0.001**	**0.342**	**<0.001**
HMGA2	Eosinophils	0.224	<0.001	0.319	<0.001
HMGA2	Neutrophils	0.127	0.003	0.279	<0.001
HMGA2	NK cells	0.196	<0.001	0.276	<0.001
HMGA2	Cytotoxic cells	0.136	0.002	0.243	<0.001
HMGA2	T cells	0.148	<0.001	0.238	<0.001
HMGA2	Th17 cells	0.145	<0.001	0.226	<0.001
HMGA2	Th2 cells	**0.240**	**<0.001**	**0.204**	**<0.001**
HMGA2	NK CD56dim cells	0.089	0.040	0.181	<0.001
HMGA2	iDC	0.016	0.722	0.126	0.004
HMGA2	T helper cells	0.092	0.034	0.121	0.005
HMGA2	B cells	0.009	0.842	0.065	0.136
HMGA2	Th1 cells	−0.017	0.699	0.041	0.352
HMGA2	CD8 T cells	0.007	0.878	−0.021	0.624
HMGA2	Mast cells	−0.027	0.540	−0.028	0.515
HMGA2	Tgd	−0.144	<0.001	−0.031	0.484
HMGA2	DC	−0.121	0.005	−0.052	0.235
HMGA2	NK CD56bright cells	−0.085	0.052	−0.073	0.095
HMGA2	Tcm	0.006	0.894	−0.073	0.092
HMGA2	Tem	0.030	0.493	−0.078	0.074
HMGA2	TReg	−0.013	0.763	−0.078	0.073
HMGA2	TFH	−0.150	<0.001	−0.093	0.032
HMGA2	pDC	**−0.177**	**<0.001**	**−0.225**	**<0.001**

Infiltration levels of macrophages, Th2 cells, and pDC with HMGA2 expression in LGG.

For another, HMGA2 had a similar correlation with immune cell infiltration in LGG (bottom in Figure 9A). Th2 cell and macrophage infiltration were also significantly increased in samples with high HMGA2 expression, and pDC cells were enriched in HMGA2 low expression patients. Nevertheless, although pDC infiltration also had no significant effect on LGG patient prognosis, the high degree of Th2 cell and macrophage infiltration led to a poor prognosis of LGG patients (*p* = 3.58E-05 and 3.11e-06) (bottom in Figure 9B). Thus, the high infiltration of Th2 cells and macrophages induced by HGMA2 can affect the prognosis of glioma patients to a certain extent, which depends on the grade of glioma.

Based on results from this study, HMGA2 has a close correlation with COL4A family members. Then, we further analyzed the effects of COL4A family members on immune infiltration in glioma (GBM results in Figures 9C, D, LGG results in Supplementary Figure S5). The outcome revealed that COL4A1 and COL4A2 effects on immune infiltration had a highly similar association with HMGA2 in both GBM and LGG patients, especially on Th2 cells, macrophages cells and pDC cells infiltration (r = 0.68, 0.65 and −0.39 for COL4A1, r = 0.66, 0.64 and −0.37 for COL4A2 in GBM). In contrast, COL4A3 and COL4A4 had an opposite correlation with Th2 cell and macrophage enrichment when compared with HMGA2 (r = −0.2, −0.23 and 0.28 for COL4A3, r = −0.2, −0.28 and 0.29 for COL4A4 in GBM). Thus, HMGA2 involved in immune infiltration in glioma may play a critical role in the effects of the COL4A family on patient prognosis.

**FIGURE 10:**
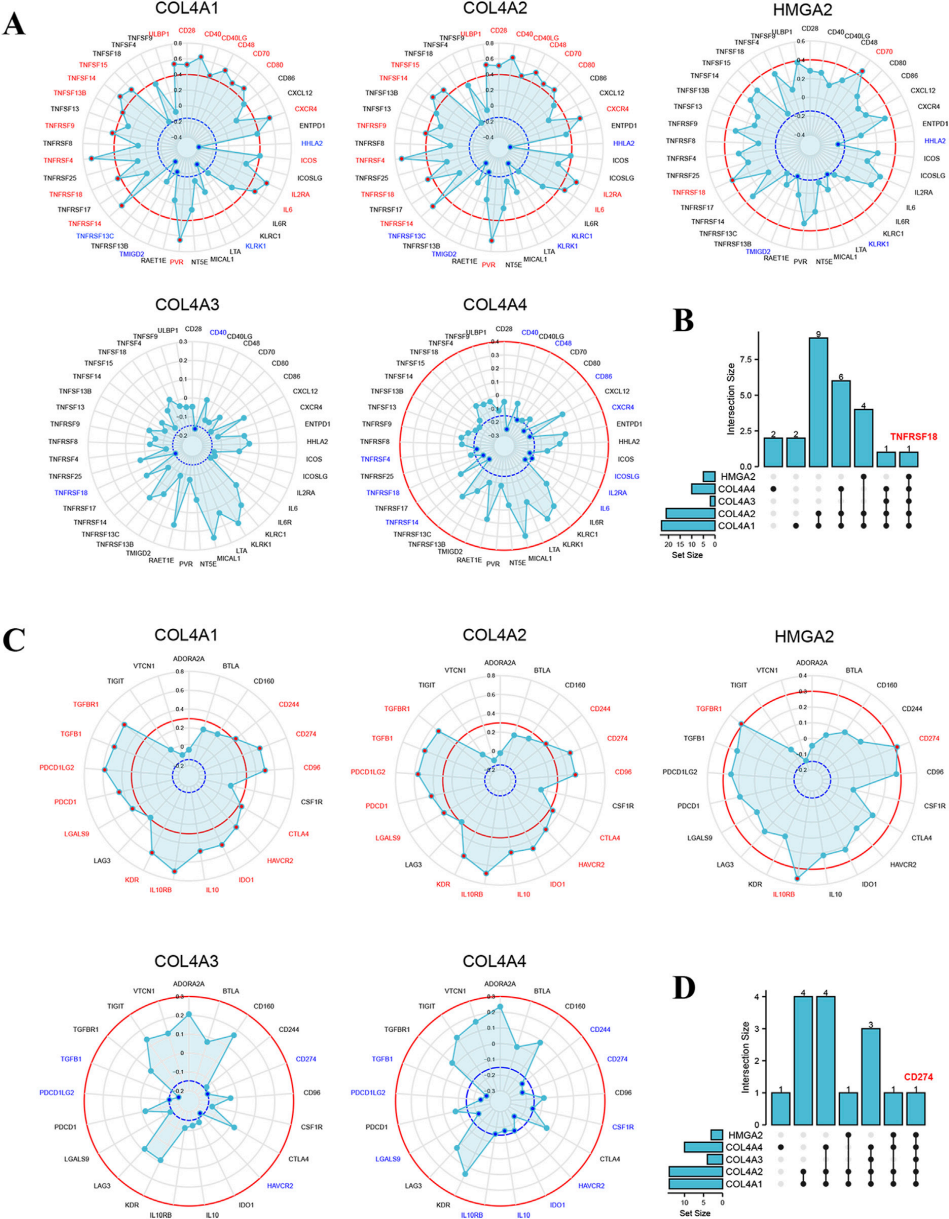
Prediction of immunomodulators associated with COL4A and HMGA2 in glioma patients **(A)**. The COL4A1-4 and HMGA2 related immunostimulators (p < 0.05) with correlation thresholds of less than −0.15 (blue) or higher than 0.4 (red) **(B)**. The overlapped immunostimulators between COL4As and HMGA2 in glioma **(C)**. The COL4A1-4 and HMGA2 correlated immunoinhibitors (p < 0.05) with thresholds of less than −0.15 (blue) or greater than 0.3 (red) **(D)**. The overlapped of COL4As versus HMGA2 corelated immunostimulators in glioma.

Moreover, to further identify the potential immunotherapy strategy of glioma targeting COL4A factors and HMGA2, immunomodulators associated with COL4A factors and HMGA2 were retrieved from the online database TISIDB (Figure 10; Supplementary Figure S6). We screened the immunostimulators with correlation thresholds of less than −0.15 or higher than 0.4 (*p* < 0.05), and the immunoinhibitors with relation thresholds of less than −0.15 or higher than 0.3 (*p* < 0.05). As shown in Figure 10 and Supplementary Table S1, 2, the immunostimulator TNFRSF18 was positively related to COL4A1, COL4A2 and HMGA2 (r = 0.436, 0.462, and 0.425, respectively), and negatively related to COL4A3 and COL4A4 (r = −0.157 and −0.207, respectively). For another, the immunoinhibitor CD274 has a positive association with COL4A1, COL4A2 and HMGA2 (r = 0.497, 0.310 and 0.315, respectively), and a negative correlation with COL4A3 and CO4A4 (r = −0.150 and −0.216). Thus, the TNFRSF18 and CD274 may have a great potential in the tumor immunotherapy of glioma.

## 4 Discussion

Collagen is critical for the function of the BM, which is a cell-associated extracellular matrix that supports tissue integrity, signaling, and barrier properties (Jayadev et al., 2019). Col IV is generated from six kinds of collagen α chains (COL4A1-6) Based on previous studies, COL4A family members have been reported to be involved in the progress of focal segmental glomerulosclerosis (Gast et al., 2016), Alport syndrome (Ozdemir et al., 2020) and cancers (Miyake et al., 2017). However, the diseases involving different COL4A members are quite different. COL4A1 and COL4A2 mutations can induce neurological diseases, including epilepsy (Zagaglia et al., 2018), hemorrhagic stroke (Jeanne et al., 2012)], and sporadic cerebral small vessel disease (Rannikmäe et al., 2015). Alterations in the COL4A3, COL4A4 and COL4A5 genes are associated with glomerular basement membrane-related diseases, such as autosomal recessive Alport syndrome (Storey et al., 2013; Hudson et al., 2003), familial focal segmental glomerulosclerosis (Andrew et al., 2014), and thin basement membrane nephropathy (Wang et al., 2004). Few studies have elucidated the function and association of different COL4A family members in the same disease until now. Therefore, when we found that the expression of different members of the COL4A family was greatly distinct in tumor tissue, especially in glioma, we speculated that the COL4A family members may have the mutually constrained effects on glioma progression.

The further analysis showed that COL4A1 and COL4A2 had similar upregulated expression levels in glioma tumor tissues. They shared a consistent association with clinicopathological parameters in glioma and a negative correlation with other members of the COL4A family. Elevated COL4A1 expression was correlated with poorer survival of patients with low-grade glioma (LGG). However, COL4A1 was predominantly expressed in stromal cells, including cancer-associated fibroblasts (CAFs) and endothelial cells. Additionally, COL4A1 expression was highly correlated with endothelial cells. Moreover, COL4A1 expression showed a strong positive relationship with marker genes for pro-tumoral immune cell infiltration, such as Tregs, M2 macrophages, and tumor-associated macrophages (TAMs) and immunosuppressive cytokine expression. COL4A1, COL4A2 could regulate the immunosuppressive microenvironment of glioma. Based on The Cancer Genome Atlas database revealed that four COL4A family members, such as COL4A1, and COL4A2, COL4A6, et al., expression are significantly upregulated in glioma tissues compared with normal nontumor tissues. However, COL4A1-2 and COL4A3-4 have completely opposite effects on glioma patient prognosis, which verified our hypothesis to a certain extent. COL4A3, COL4A4, and COL4A5 are predominantly expressed in and critical for maintaining the structural integrity of the glomerular basement membrane, alterations in these genes lead to basement membrane disruption, which is the primary cause of Alport syndrome (Savige et al., 2022).

To account for this interesting phenomenon, we found a potential explanation in previous reports. Compared with the major Col IV composed of two α1 and one α2 chains, the minor Col IV composed of α3, α4, α5 chains or two α5 and one α6 chains has distinct biomechanical properties. The former is the most abundant component of nearly all basement membranes, especially in the vascular endothelium and vascular smooth muscle (Kuo et al., 2012), while the networks formed by the latter have a much greater density of disulfide interchain crosslinks (Cosgrove and Liu, 2017). This means that the minor Col IV network composed of α3-α6 chains may be more compact, stable and resistant to proteolytic degradation than the major Col IV network. In addition, Col IV was confined to pial-glial membranes and thickened vessel walls in glioma (Rd and Dd, 1985). Col IV staining indicated that pial-glial membranes remained relatively intact and that the number of branching capillaries was significantly increased in low-grade glioma, while disruption of pial-glial membranes and vascular glomeruloid proliferation were observed in highly invasive glioblastomas (K et al., 1989). Therefore, the increased vessel formation in tumors may be associated with the upregulation of COL4A1 and COL4A2 expression, and the aggressiveness of the tumor may result in the downregulation of COL4A3 and COL4A4 expression and minor Col IV degradation to promote invasion of tumors.

However, although there was a close connection between COL4A family members and glioma patient survival in univariate Cox regression analysis, the multivariate Cox regression analysis results indicated that except for COL4A6, the rest of the genes could not function as independent prognostic factors for glioma patient prognosis. It suggested that the COL4As may influence the prognosis of glioma patients in an indirect way. To further investigate this potential synergistic factor, the functions and interaction network of the top DE-ceRNAs associated with COL4As expression were further identified (Cao et al., 2021a; Cao et al., 2021b). We found the top DE-ceRNAs were highly enriched in the processes of“tumor development” and “immune regulation”. And nine hub-DE-ceRNAs were sorted out from the triple regulatory network, including two lncRNAs, two miRNAs and five mRNA. By determining the base pairing pattern, we obtained the COL4As-H19/HOTAIR-miR148a/miR222-HMGA2 axis from the hub-network, which proposes that HMGA2 is a coregulated target molecule by COL4A members. Our novel finding in glioma reveals that HMGA2 is regulated by COL4As, particularly COL4A1, although this regulatory relationship has been previously reported in other malignanciesincluding esophageal carcinoma (ESCA), stomach adenocarcinoma (STAD), hepatocellular carcinoma (LIHC), and colon adenocarcinoma (COAD) (Tang et al., 2024).

HMGA2 is a transcriptional regulator involved in the cell cycle, cell division, growth regulation, mitosis, transcription, and transcription regulation (Zhang et al., 2019). It has been reported to participate in many biological processes in tumors, such as epithelial-to-mesenchymal transition (Dong et al., 2017), angiogenesis (Li Y. et al., 2020), and cancer cell proliferation (Li et al., 2014). HMGA2 is widely recognized as a novel oncogene that significantly influences tumor initiation, progression, and prognosis. *In vitro*, studies demonstrate that HMGA2 knockdown can suppresses glioma cell migration, invasion, and proliferation. These collectively indicated that HMGA2 promotes malignant progression in gliomas. Notably, we observed elevated HMGA2 expression levels in glioma tissues. Furthermore, high HMGA2 expression serves as an independent prognostic factor for poor survival in glioma patients. An additional study indicated that HMGA2 is a potential IDH-independent poor prognostic biomarker for glioma patients. Its overexpression leads to the acceleration of cell migration and invasion in malignant gliomas, thereby expediting their progression (Zhang et al., 2018). In the present study, we found that HMGA2 was also mainly involved in proliferation (PI3K-Akt pathway), invasion and migration (protein digestion and absorption and ECM-receptor interaction) in glioma, which is consistent with the function of the COL4A family. Further Cox regression analysis suggested that HMGA2 can function as an independent prognostic factor for glioma patient prognosis. Thus, HMGA2 may be a critical factor for COL4A family members involved in glioma progression and a potential therapeutic target of glioma.

In addition, HMGA2 was reported to serve as a driver of inflammation and further be involved in hypermethylation-induced acute liver injury (H et al., 2017) and corneal epithelial cell inflammation (Li X. et al., 2020), which was also consisted with the potential function of COL4As related DE-ceRNAs we presented in Figure 5. As known, immune infiltration in tumors also have significant effect on the prognosis of the patient (Jin et al., 2020). Therefore, by using immune infiltration analysis, we found HMGA2 has an association with Th2 cells, macrophages and pDCs, in glioma. And this correlation can also be observed in COL4A1 and COL4A2 and was the opposite in COL4A3 and COL4A4. It further demonstrated a potential regulatory relationship between COL4As and HMGA2. HMGA2 in cancer cells can enhance macrophages recruitment both *in vitro* and *in vivo* conditions. Mechanistically, HMGA2 directly binds to the STAT3 promoter to activate its transcription, subsequently inducing CCL2 secretion which can facilitate macrophage recruitment (Wang et al., 2022). Additionally, HMGA2 promotes tumor progression by regulating macrophage proliferation, migration, polarization and angiogenesis via CXCL12/CXCR4-dependent mechanisms. *In vivo*, studies demonstrated that HMGA2-mediated regulation of macrophage polarization through the CXCL12/CXCR4 axis significantly promotes tumor metastasis (Cheng et al., 2021).

For another, based on the results from Figure 10, two immunomodulators were correlated with COL4As and HMGA2, including immunostimulator TNFRSF18 and immunoinhibitor CD274, which are also known as PDL-1 and GITR and have important effects on prognosis of glioma (Li Y. et al., 2020; Li et al., 2014; Zhou et al., 2021). Besides, the PDL-1 was reported to significantly express in tumor-associated macrophages (TAMs) which related to a poor prognosis in cancers (Zhang et al., 2018; H et al., 2017; Dammeijer et al., 2020; Du et al., 2020). And the GITR involved in the regulation of immunological homeostasis by Treg cells and pDCs (Yang et al., 2017). The activation of GITR leads to an alleviation in Treg cell-mediated suppression of anti-tumor immune response and an activation of NK cells (Hanabuchi et al., 2006), inducing potent anti-tumor effector cells in GBM (Amoozgar et al., 2021). Therefore, combining with the previous results, we concluded that the PDL-1 and GITR function-related immune cells in glioma were also highly associated with COL4As and HMGA2, which further confirmed the effects of COL4As and their target factor HMGA2 on glioma progress.

## 5 Conclusion

In general, the presented study provided an overview of the association of COL4A family members with glioma progression and present a COL4A-H19/HOTAIR-miR148a/miR222-HMGA2 axis in glioma established by a COL4A-related ceRNA interaction network. Additionally, we further revealed that HMGA2 can be a novel significant prognostic biomarker in glioma and function as a potential therapeutic target for glioma.

## Data Availability

The original contributions presented in the study are included in the article/Supplementary Material, further inquiries can be directed to the corresponding authors.
