# Supplementary material for: Prognostic significance and multivariate modeling of COL4A family genes and HMGA2 in glioma
Source: Front Pharmacol. 2025 Apr 25;16:1591932. doi: 10.3389/fphar.2025.1591932 (PMC12062008; doi:10.3389/fphar.2025.1591932)
Supplement: Supplementary file 4 [file DataSheet1.pdf]

Supplementary Material

Supplementary Data

1 Methods

1.1 Correlation gene screening and interaction network construction

The genes associated with COL4A members and HMGA2 were sorted from the GBM and LGG cohorts in the cBioPortal dataset. Correlation analysis of target factors with associated genes or differentially expressed ceRNAs was performed using Spearman's test and visualized by the 'ggplot2' package in R. The PPI network of the top 100 correlated genes was analyzed by the STRING database and visualized by Cytoscape v3.7.1 software. The interaction networks were represented as graphs with nodes depicting proteins, edges illustrating associated interactions and the node size corresponding to the connection degree. By using the plug-in 'cytoHubba', we further identified the highly connected nodes (degree >= 30 in the correlation gene network) and created a hub network.

1.2 Functional enrichment analysis

To understand the potential biological processes and pathways of COL4As- and HMGA2-associated genes in interaction networks and the differentially expressed ceRNAs in the triple regulatory network, we conducted functional enrichment analysis for those factors. GO enrichment (including BP, CC, and MF) was performed by the 'TopGO' package, and KEGG pathway analysis was performed by the 'clusterprofiler' package in R. An adjusted $p < 0.05$ was determined to be statistically significant. The bubble plots and GO/KEGG network plots for the top 4 GO and top 5 KEGG terms were visualized using the 'ggplot2' and 'clusterProfiler' packages, respectively.

1.3 Patients' survival analysis

After assessing the GBM and LGG cohorts in the TCGA clinical datasets, 695 glioma patients were included in the survival analysis. Three clinical survival outcome endpoints were chosen for the clinical endpoint analysis: OS, progression-free interval (PFI) and disease-specific survival (DSS). By using the 'survival' package with overall survival in R, we performed univariate and multivariate Cox regression analysis to appraise the association of the COL4As and HMGA2 expression signature with clinical characteristics (including age, sex, WHO grade, IDH status, 1p/19q codeletion and histological type). Depending on the univariate Cox model analysis of COL4As and the 9 hub ceRNAs, Kaplan-Meier survival curves were drawn and generated by the 'survminer' package with log-rank p, hazard ratio (HR) and 95% confidence interval (CI) values reported. The nomograms were developed using multivariable Cox models and were generated with the 'rms' package. Receiver operating characteristic (ROC) curves and calibration plots were used to evaluate the performance of the nomogram.

1.4 Correlation analysis of clinicopathological characteristics

Based on the clinicopathological characteristics data and COL4A gene expression data of patients from the GBM and LGG cohorts in TCGA database, we analyzed the correlation between the COL4A expression level and clinicopathological features. The WHO grade and histological type were divided into three and four subgroups, respectively. A Kruskal−Wallis test followed by Dunn’s multiple comparison test was used for comparison of COL4A mRNA expression and those subgroups. The association of COL4A mRNA expression with p/19q codeletion, IDH status and primary therapy outcome was calculated via the Mann-Whitney-Wilcoxon test. $p < 0.05$ was considered significant.

We further used ROC curves to compare the specificity and sensitivity of the six COL4A member expression levels to different clinicopathological features and calculated their cutoff values. The area under the curve (AUC) and its 95% CI were determined by using the ‘pROC’ package, and AUCs of ROC curves for different clinicopathological characteristics were compared and visualized by the ‘ggplot2’ package.

1.5 The ceRNA interaction and regulation network in glioma patients

Based on the RNA-Seq data from the TCGA datasets, patients with GBM and LGG were divided into COL4Ashigh and COL4Aslow expression groups by the median values of COL4A mRNA expression. The differentially expressed ceRNAs in COL4Ashigh and COL4Aslow glioma samples were analyzed by the ‘DESeq2’ package in R. We determined the differential expression ceRNAs with thresholds of $|logFC| > 1$ and $p < 0.05$, volcano plots of the ceRNAs were generated using the ‘EnhancedVolcano’ package, and expression heatmaps were visualized by the ‘pheatmap’ package. The top 10 differentially expressed ceRNAs (including lncRNAs0, miRNAs, and mRNAs) were selected to build the interaction network. To investigate the interaction between ceRNAs, the LncBase Predicted v.2 (http://carolina.imis.athena-innovation.gr/diana_tools/web/index.php?r=lncbasev2%2Findex-predicted) and LncACTdb 2.0 datasets (http://bio-bigdata.hrbmu.edu.cn/LncACTdb/) were used to predict the differentially expressed lncRNA-miRNA interaction pairs and the binding target sequence. The starBase (http://starbase.sysu.edu.cn), miRbase (http://www.mirbase.org) and TarBase (http://carolina.imis.athena-innovation.gr/diana_tools/web/index.php?r=tarbasev8/index) datasets were used to predict differentially expressed miRNA-mRNA interaction pairs and the miRNA targeted sequence in the 3’UTR of mRNAs. After integrating the ceRNA interaction pairs, the lncRNA-miRNA-mRNA triple regulatory network was visualized by Cytoscape software. Then, ceRNAs with connection degree ≥ 2 and correlated to at least one node of each different type of ceRNA were further selected to generate the hub-regulation network.

1.6 Immunohistochemistry staining image of HMGA2

The immunohistochemistry images of the protein expression of HMGA2 between normal and GBM samples were directly visualized by the HPA dataset1. (http://www.proteinatlas.org).

1.7 Immune infiltration analysis of COL4As and HMGA2

To investigate the association of the expression of target genes and tumor-infiltrating immune cells, we conducted the ‘GSVA’ package in R to measure the correlation of COL4As and HMGA2 expression with 24 tumor-infiltrating immune cells, including Th2 cells, macrophages, T helper cells, neutrophils, pDCs, eosinophils, cytotoxic cells, CD4 T cells, NK CD56dim cells, iDCs, Th17 cells,

NK cells, Tgd cells, CD8 T cells, B cells, Th1 cells, DCs, Tem cells, mast cells, and Tcm cells, TFH cells, Treg cells, pDCs, NK CD56bright cells, in the GBM and LGG cohorts.

Furthermore, we analyzed the correlation of COL4As and HMGA2 with the markers of three tumor-infiltrating immune cells, Th2 cells, macrophages and pDCs in GBM and LGG. The prognostic value of three tumor-infiltrating immune cells in GBM and LGG was evaluated by the TIMER online dataset (https://cistrome.shinyapps.io/timer/). Immunoinhibitors and immunostimulators that were significantly correlated with HMGA2 regarding gene expression with thresholds of $p < 0.05$ and rho>0.2 in both LGG and GBM were selected from the TISIDB dataset (http://cis.hku.hk/TISIDB/index.php).

1.8 Analysis of single-cell RNA-seq

The GBM single-cell RNA-seq data were obtained from the GEO database GSE117891. The gene expression matrix was converted to Seurat objects using the Seurat R package (Version 3.0.2). UMI counts were transformed into log-space after the aforementioned trimming steps. Cells were removed when the number of detected genes was less than 200. The top 2,500 highly variable genes were selected for further clustering analysis. After scaling the data, PCA was performed based on the highly variable genes. The first 50 principal components were chosen to further reduce dimensionality using the t-distributed statistical neighbor embedding (t-SNE) algorithm. Each cluster was then annotated with the common cell markers. After determining the cell subpopulations, we mapped and visualized the COL4As and HMGA2 genes onto the cell subsets to identify the distribution of target genes in different cell subpopulations.

2 Supplementary Figures

Supplementary figure legend

Supplementary Figure 1. The prognostic model for the COL4As member in glioma (GBM and LGG). Univariate (A) and multivariate (B) Cox regression analyses for COL4A expression and glioma-related clinical features. C. The correlation between the six members of COL4As expression and risk score in the prognostic model. The efficiency comparation between the COL4As-related risk model and clinical features model by K-M curves (D), diagnostic ROC and DCA curve (E), and time dependent-ROC curve (F) in the TCGA glioma cohorts (GBM and LGG).

Supplementary Figure 2. The heatmap of top 10 DE-mRNAs, DE-lncRNAs and DE-miRNAs related to COL4As expression. The red marker represented the upregulation, and blue makers represented downregulation.

Supplementary Figure 3. The correlation of hub-ceRNAs between COL4A1-2 and COL4A3-4, respectively.

Supplementary Figure 4. COL4A and HMGA2 enrichment analysis in single-cell sequencing data. (A) and(B). Dimensional-reduction clustering and cluster identification for the single-cell sequencing data in GSE117891. (C) and (D). Enrichment analysis of COL4As and HMGA2 in different clusters.

Supplementary Figure 5. The immune infiltration analysis of COL4As in LGG patients.

Supplementary Figure 6. Prediction of immunomodulators associated with COL4A5 and COL4A6 in glioma patients. (A). The COL4A5 and COL4A6 related immunostimulators ($p < 0.05$) with correlation thresholds of less than -0.15 (blue) or higher than 0.4 (red). (B). The COL4A5 and COL4A6 correlated immunoinhibitors ($p < 0.05$) with thresholds of less than -0.15 (blue) or greater than 0.3 (red).
