# Supplementary figures and images for: Prognostic significance and multivariate modeling of COL4A family genes and HMGA2 in glioma
Source: Front Pharmacol. 2025 Apr 25;16:1591932. doi: 10.3389/fphar.2025.1591932 (PMC12062008; doi:10.3389/fphar.2025.1591932)

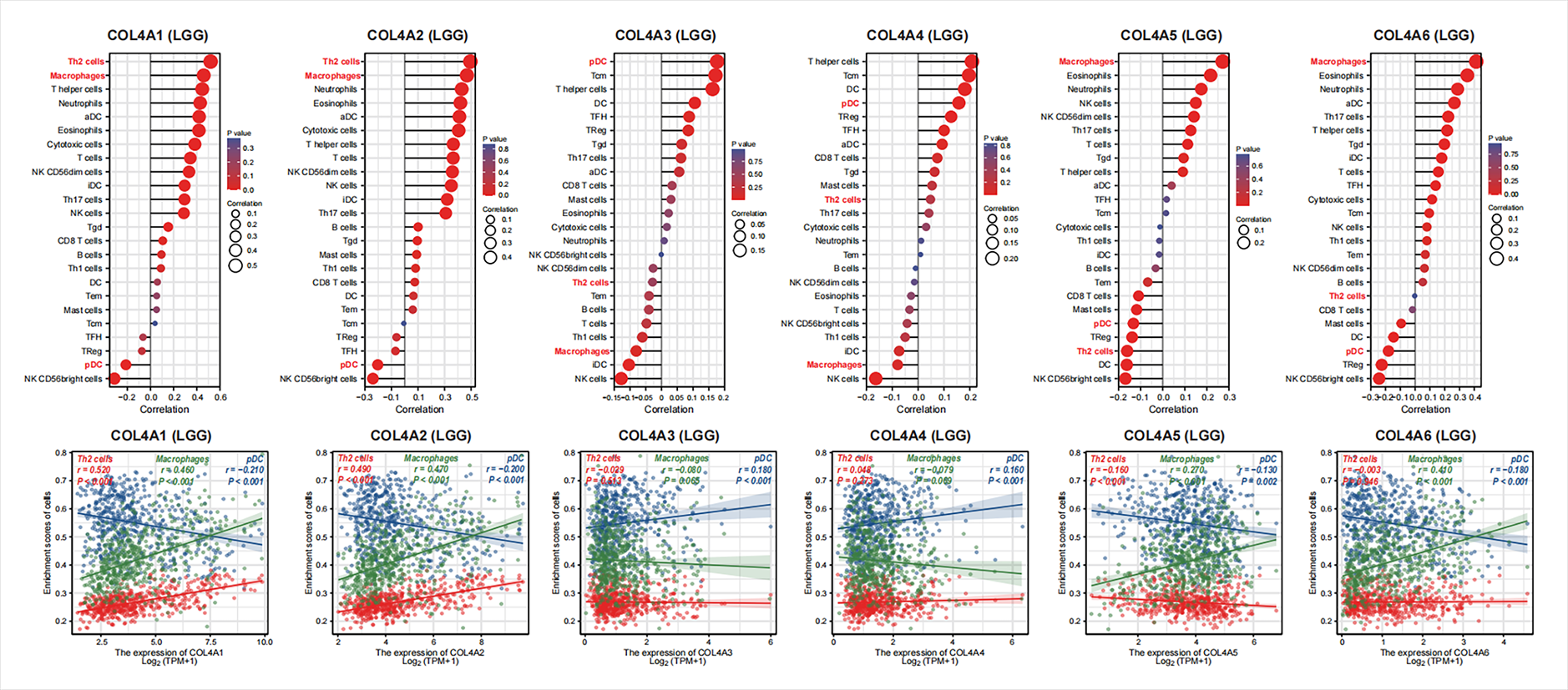

Supplement: Supplementary file 5 [file DataSheet2.zip › Figure S5.tif]

A

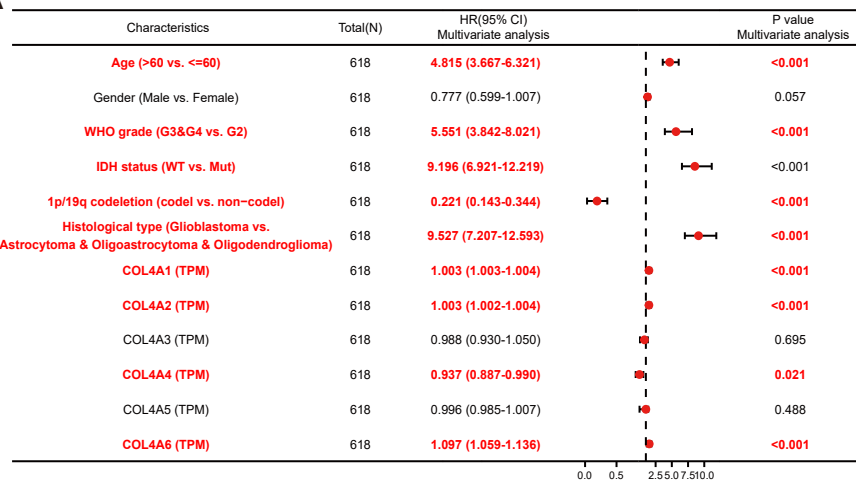

B

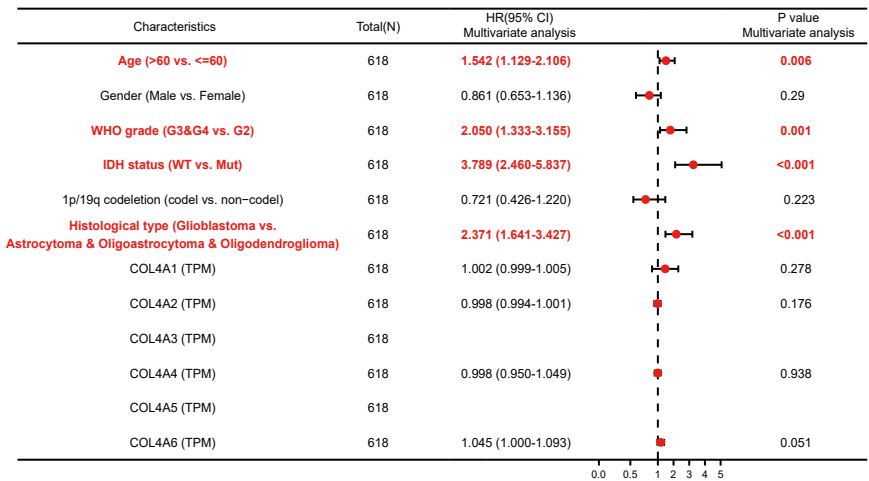

C

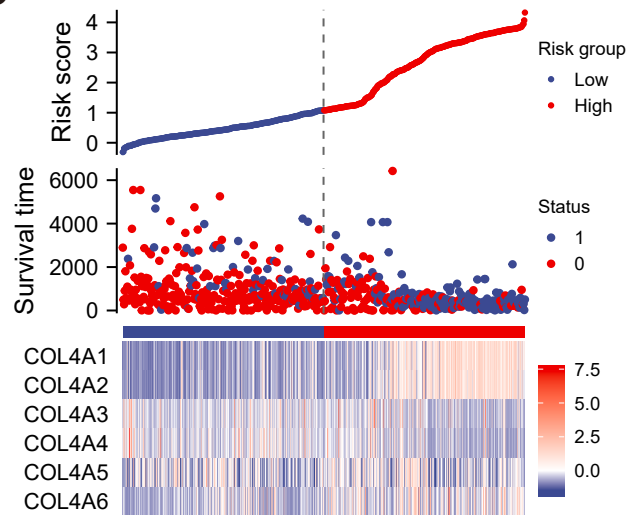

D

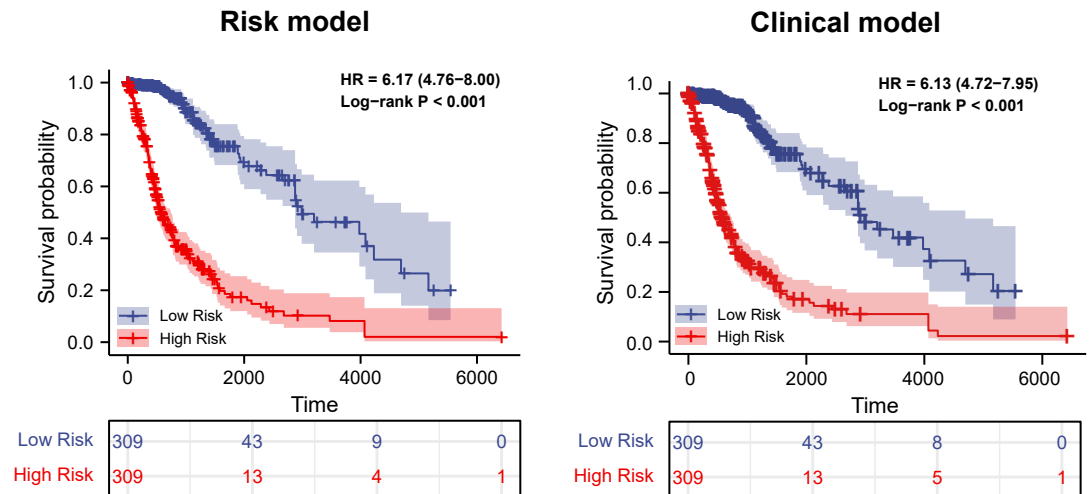

E

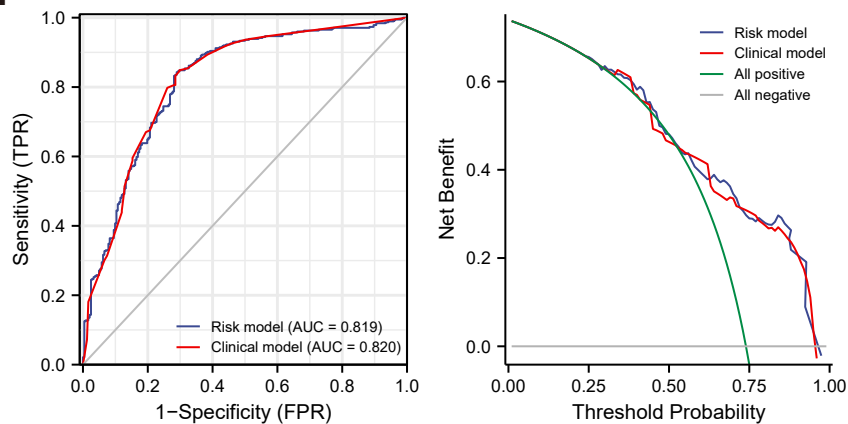

F

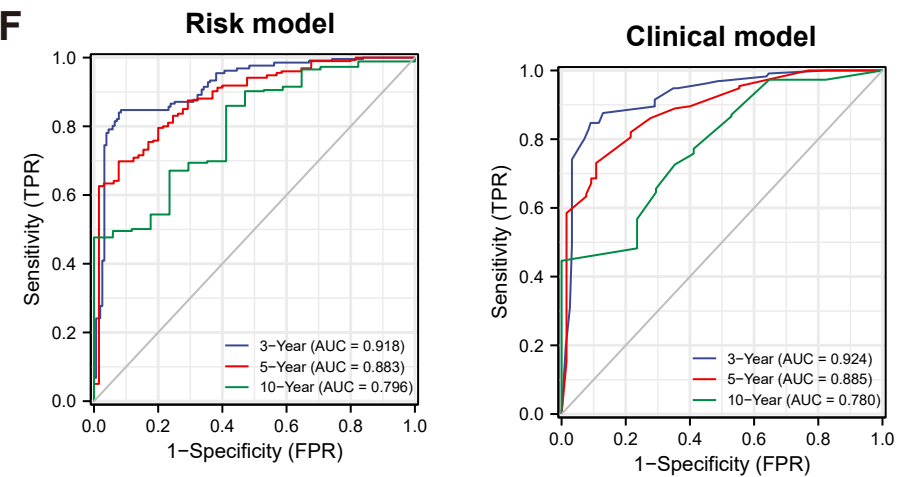

Supplement: Supplementary file 5 [file DataSheet2.zip › figure S1.pdf]

COL4A1

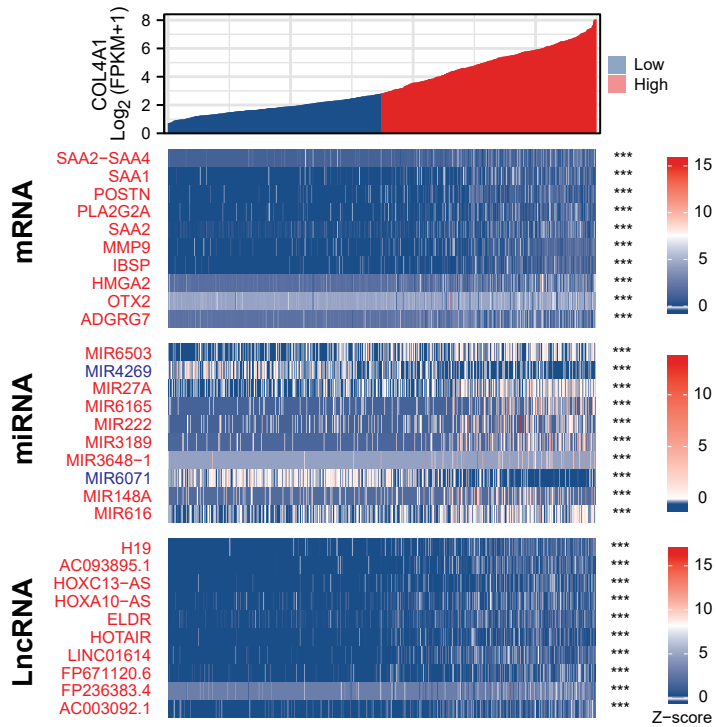

COL4A2

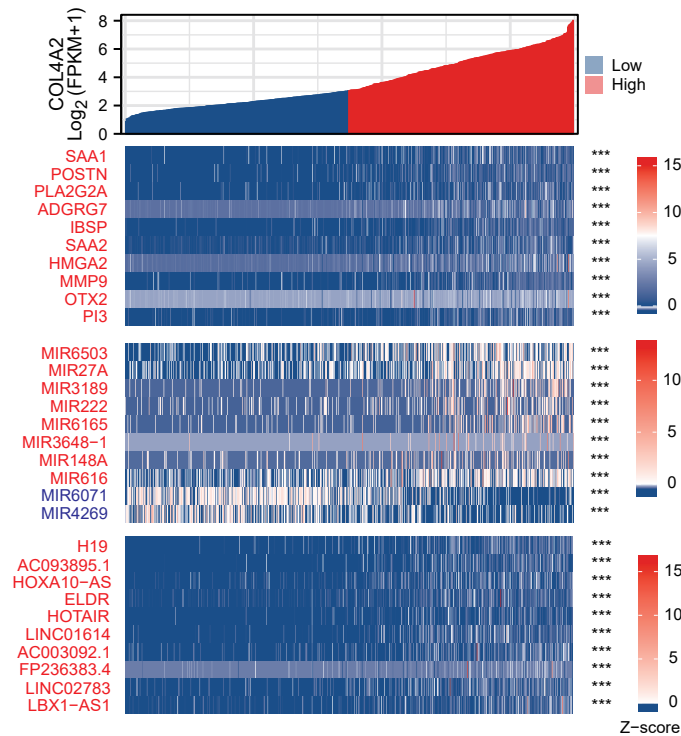

COL4A3

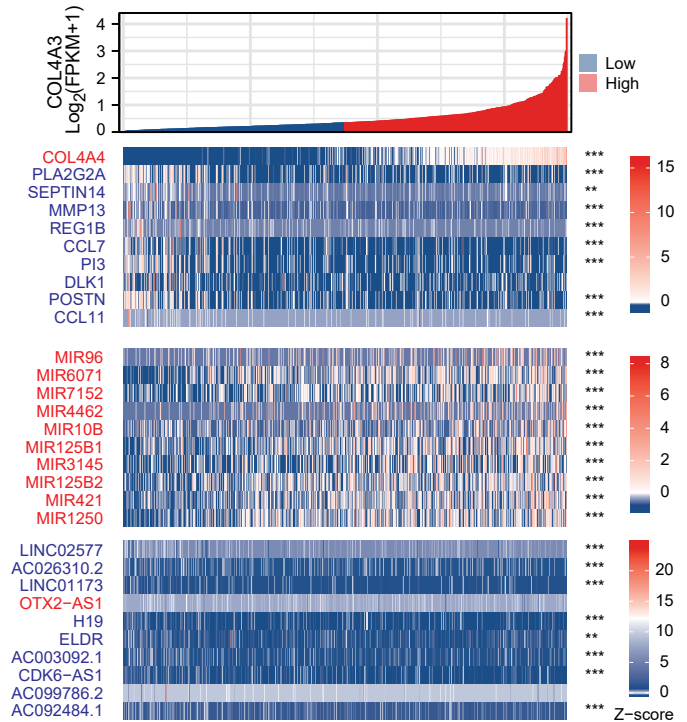

COL4A4

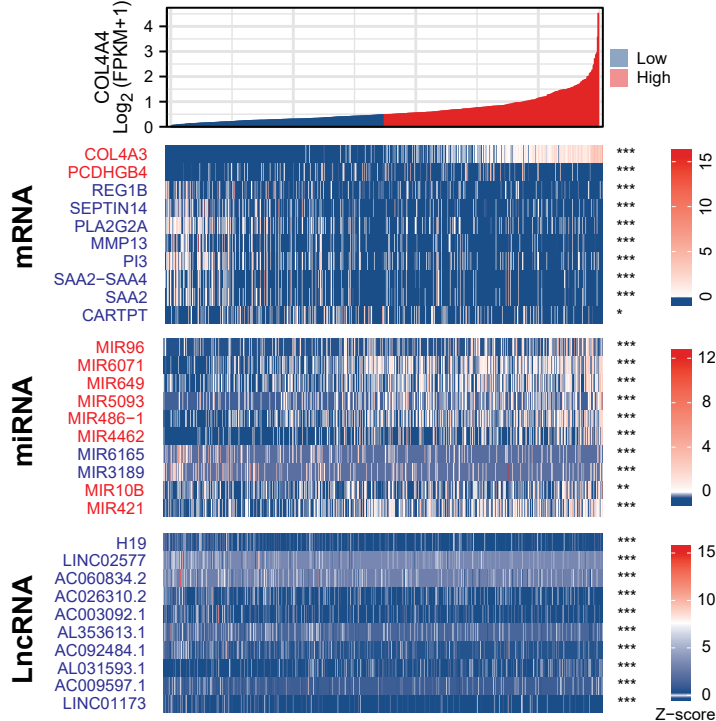

COL4A5

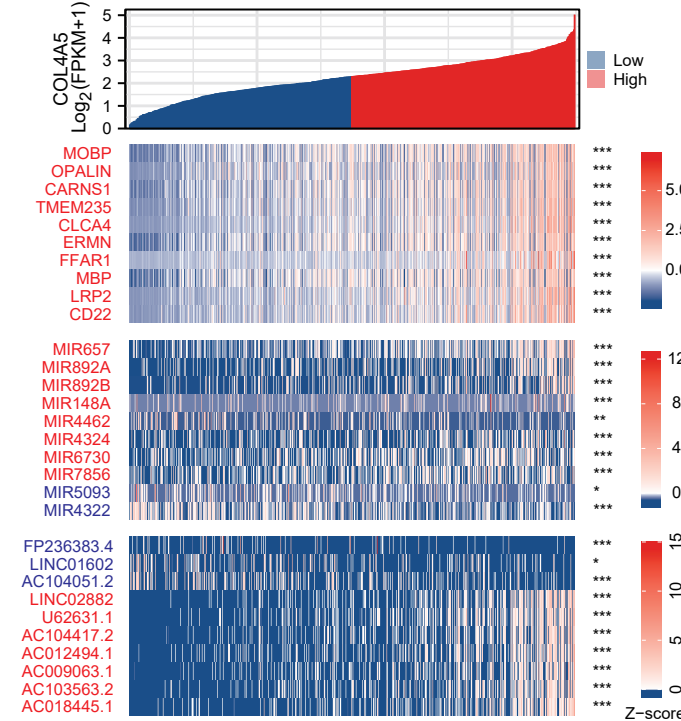

COL4A6

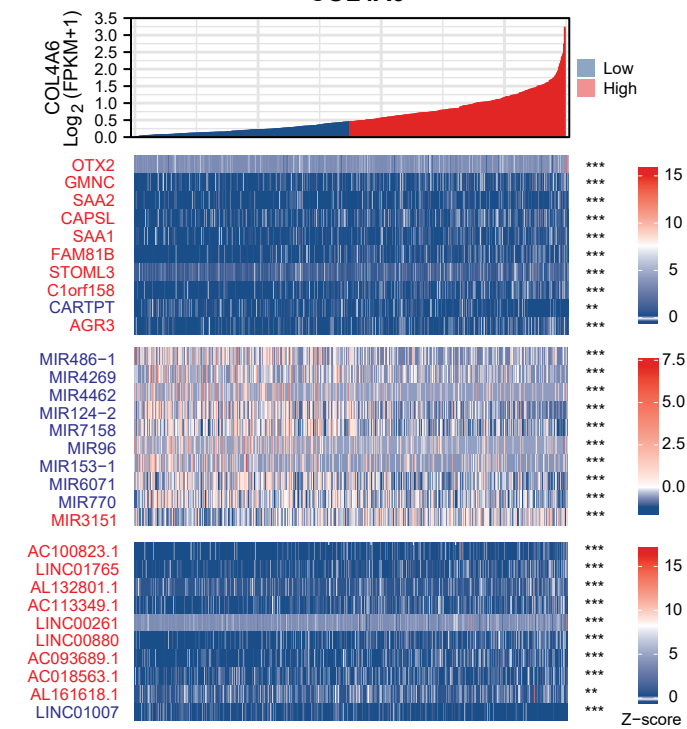

Supplement: Supplementary file 5 [file DataSheet2.zip › figure S2.pdf]

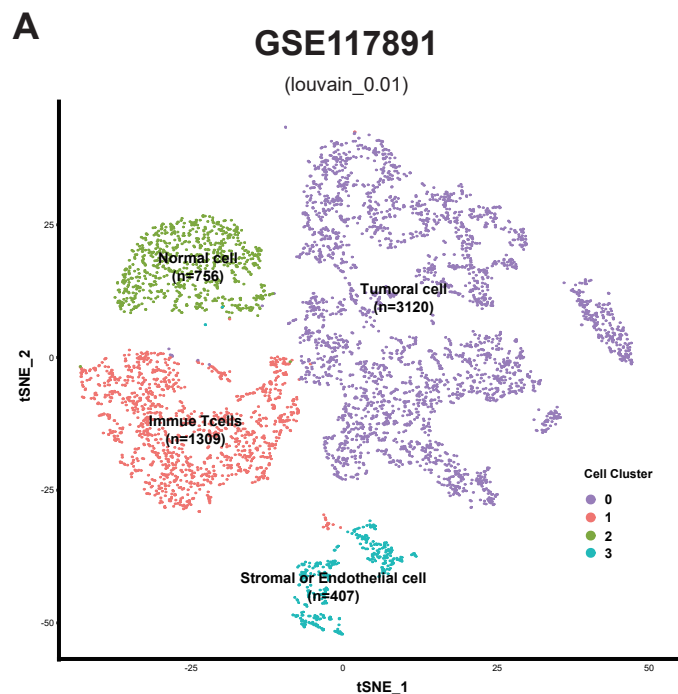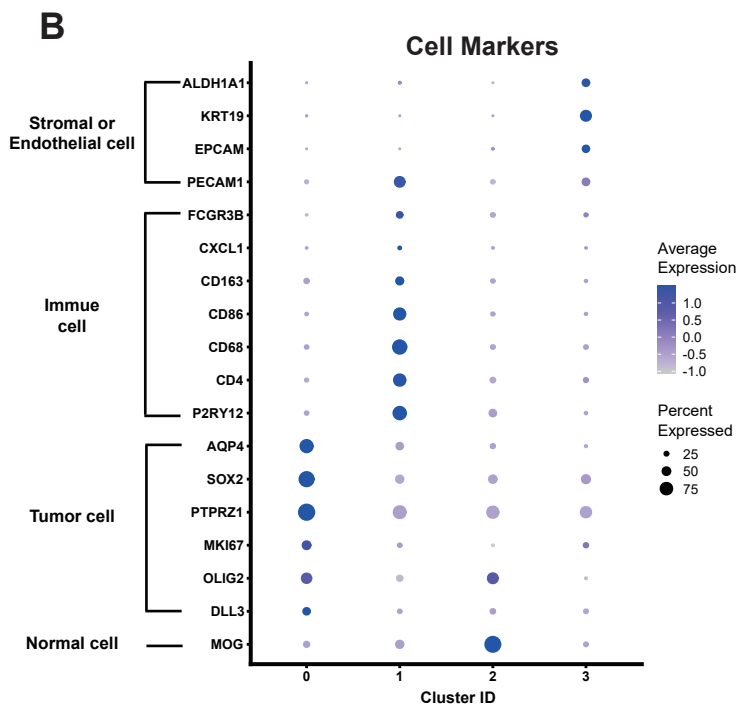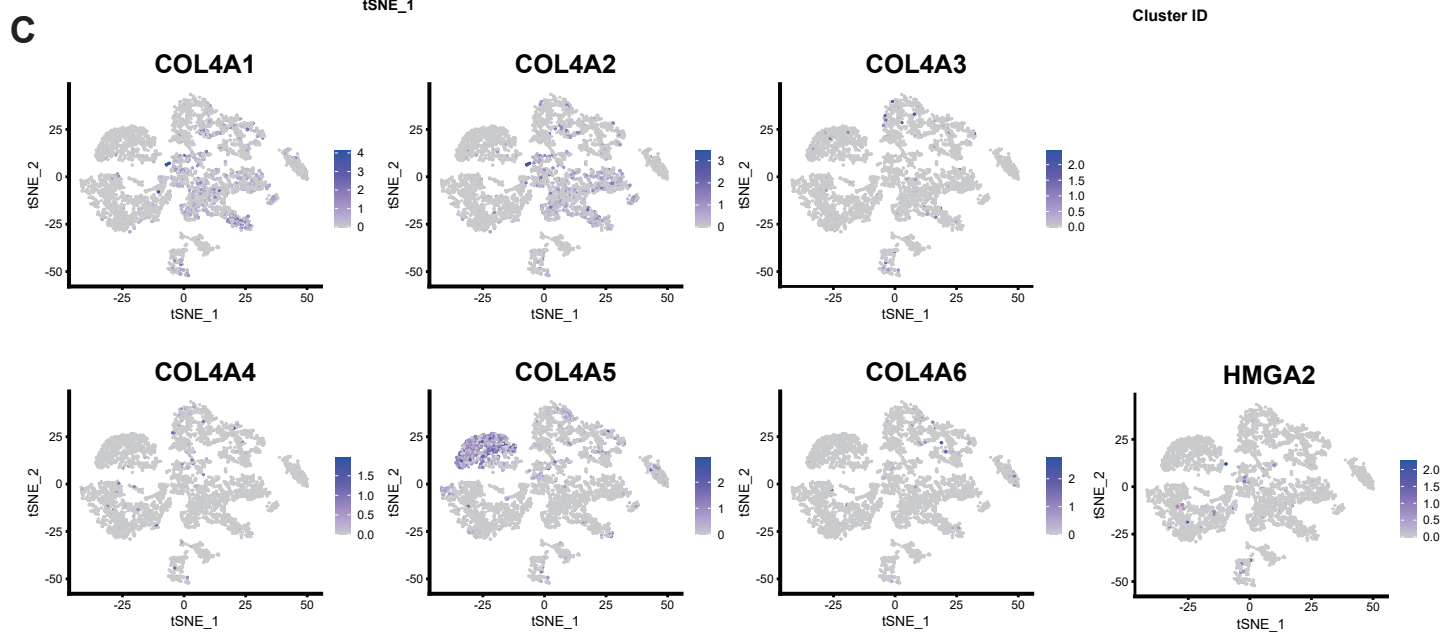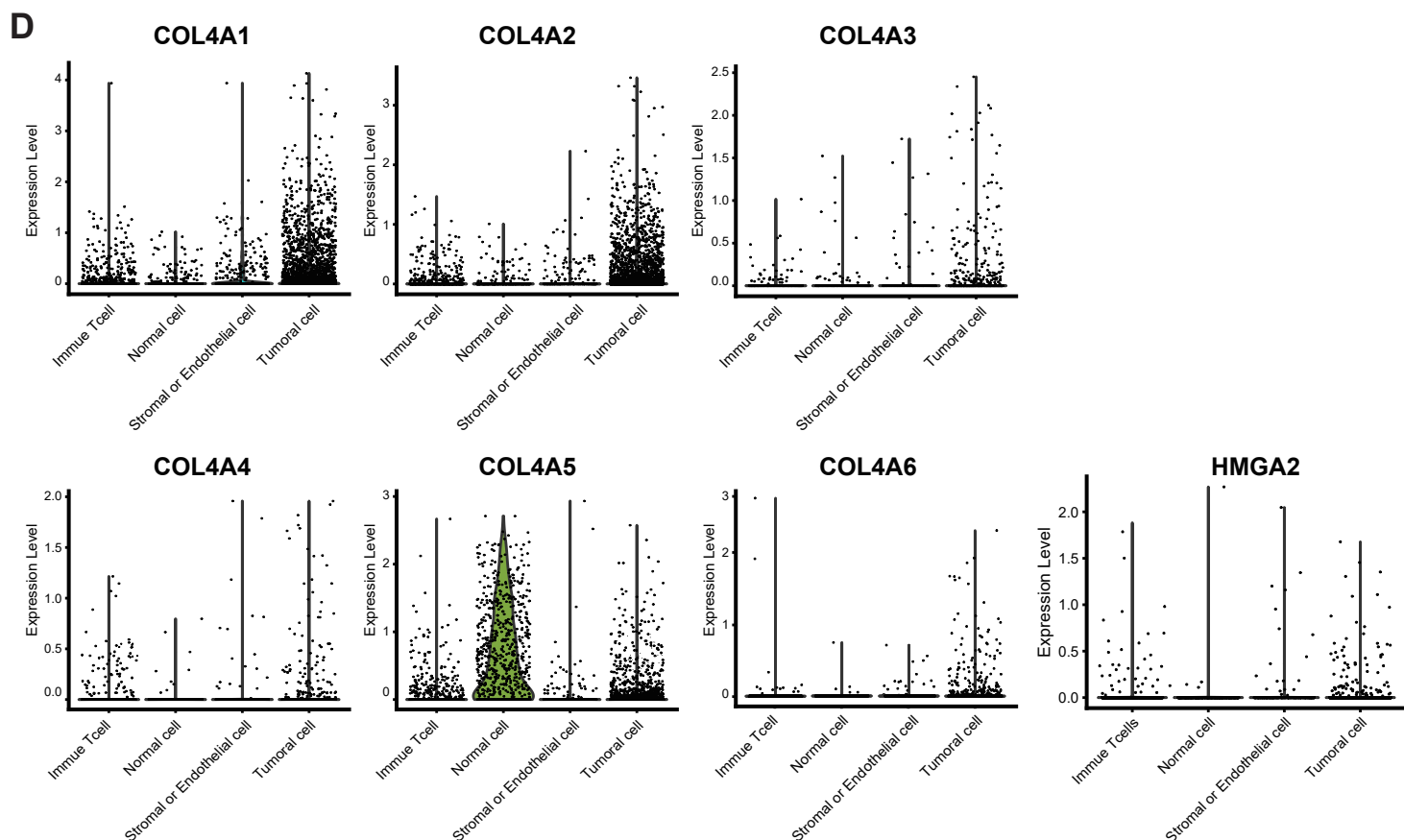

Supplement: Supplementary file 5 [file DataSheet2.zip › figure S4.pdf]

# Immunostimulator

## COL4A5

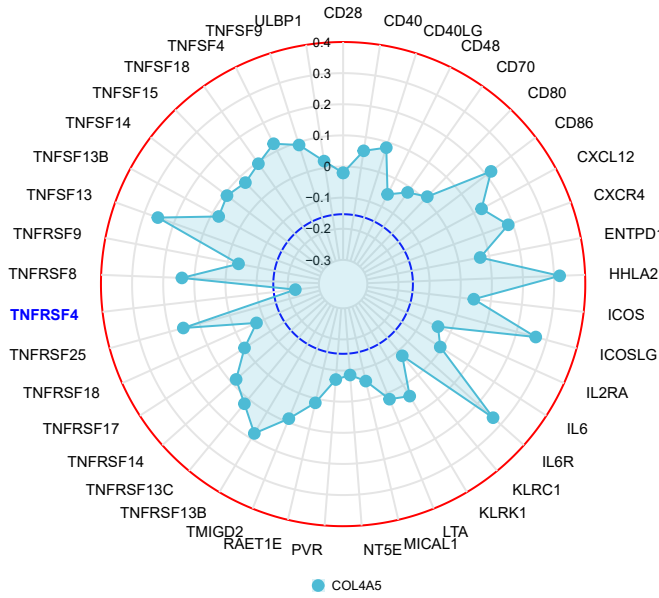

## COL4A6

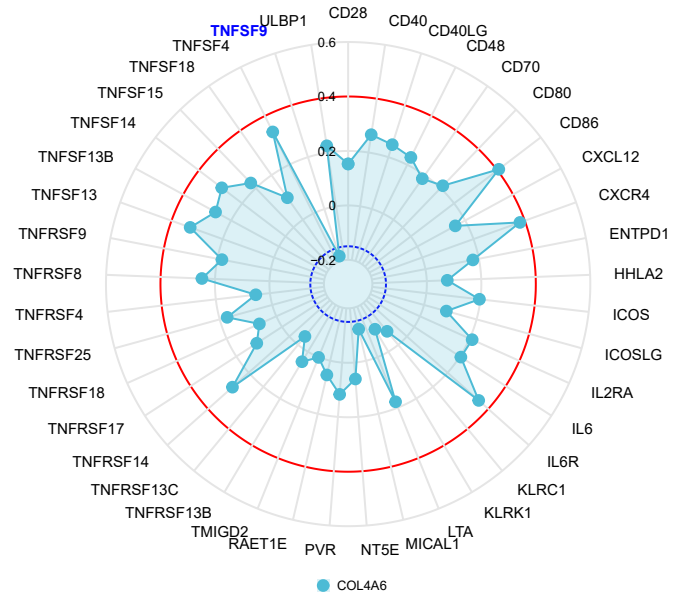

# Immunoinhibitor

## COL4A5

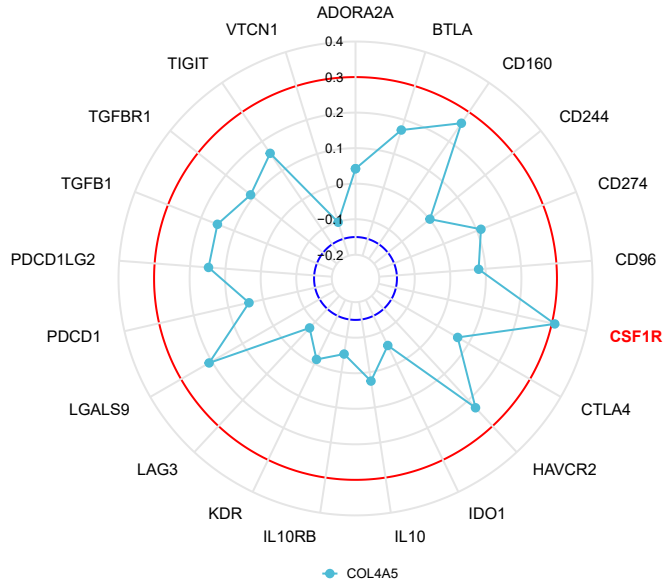

## COL4A6

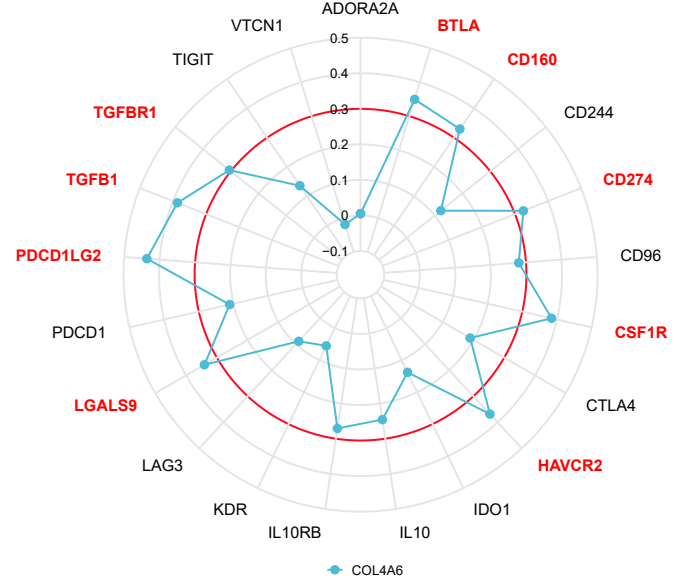

Supplement: Supplementary file 5 [file DataSheet2.zip › figure S6.pdf]

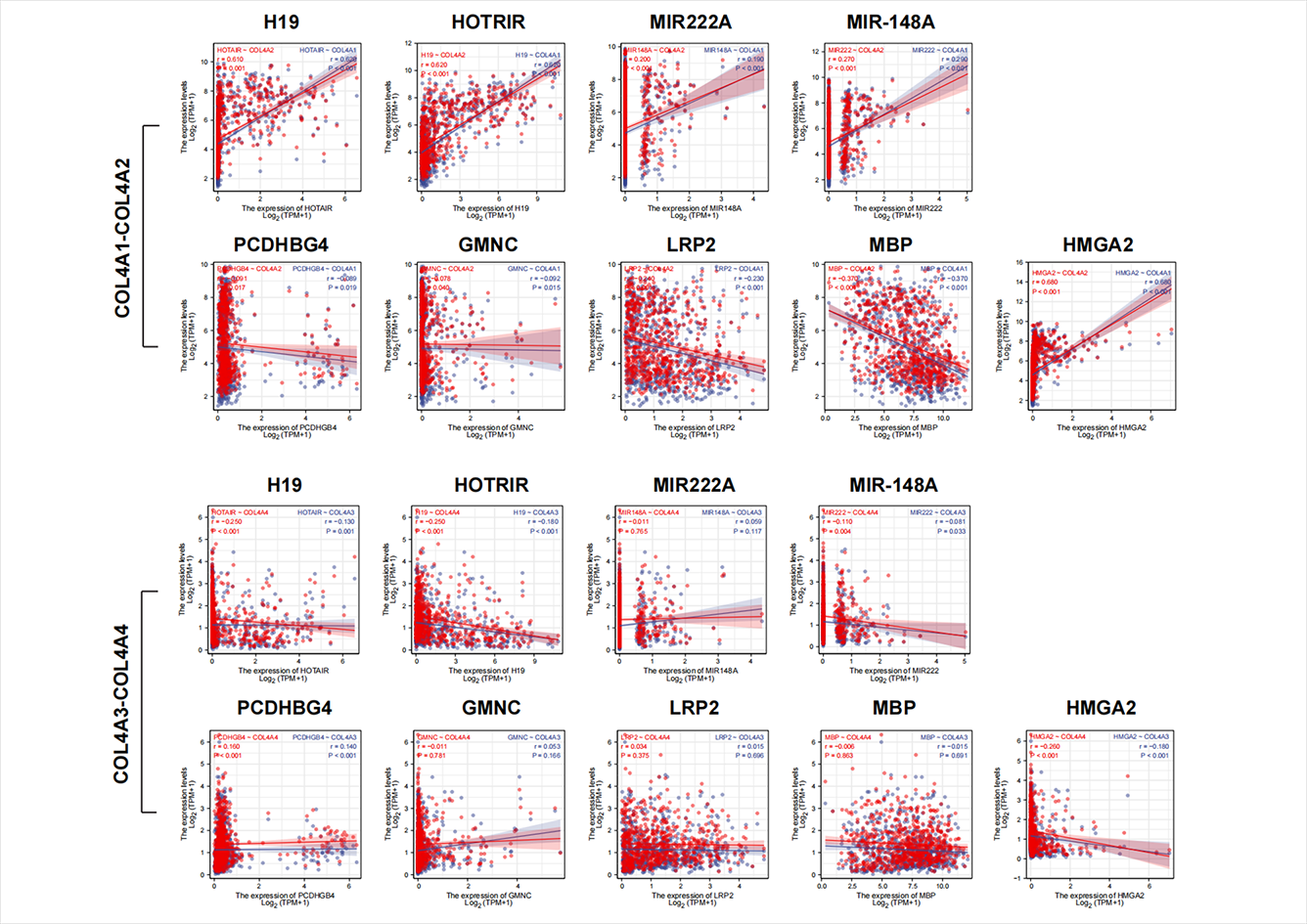

Supplement: Supplementary file 5 [file DataSheet2.zip › Figure S3.tif]
